# Nucleotide ecto-enzyme metabolic pattern and spatial distribution in calcific aortic valve disease; its relation to pathological changes and clinical presentation

**DOI:** 10.1007/s00392-019-01495-x

**Published:** 2019-05-29

**Authors:** Barbara Kutryb-Zajac, Patrycja Jablonska, Marcin Serocki, Alicja Bulinska, Paulina Mierzejewska, Daniela Friebe, Christina Alter, Agnieszka Jasztal, Romuald Lango, Jan Rogowski, Rafal Bartoszewski, Ewa M. Slominska, Stefan Chlopicki, Jürgen Schrader, Magdi H. Yacoub, Ryszard T. Smolenski

**Affiliations:** 1 Department of Biochemistry, Medical University of Gdansk, Dębinki 1 Street, 80-211 Gdańsk, Poland; 2 Department of Biology and Pharmaceutical Botany, Medical University of Gdansk, Hallera 107 Street, 80-416 Gdańsk, Poland; 3 Department of Molecular Cardiology, Heinrich-Heine-University Düsseldorf, Universitätsstr. 1, 40225 Düsseldorf, Germany; 4 Jagiellonian Centre for Experimental Therapeutics, Bobrzyńskiego 14 Street, 30-348 Kraków, Poland; 5 Department of Cardiac Anesthesiology, Medical University of Gdansk, Dębinki 7 Street, 80-211 Gdańsk, Poland; 6 Chair and Clinic of Cardiac and Vascular Surgery, Medical University of Gdansk, Dębinki 7 Street, 80-211 Gdańsk, Poland; 7 Heart Science Centre, Imperial College of London at Harefield Hospital, Harefield, Middlesex UB9 6JH UK

**Keywords:** Calcific aortic valve disease, Ecto-5′-nucleotidase, Ecto-nucleoside triphosphate diphosphohydrolase 1, Adenosine deaminase, Adenosine, Adenosine receptors

## Abstract

**Background:**

Extracellular nucleotide metabolism contributes to chronic inflammation, cell differentiation, and tissue mineralization by controlling nucleotide and adenosine concentrations and hence its purinergic effects. This study investigated location-specific changes of extracellular nucleotide metabolism in aortic valves of patients with calcific aortic valve disease (CAVD). Individual ecto-enzymes and adenosine receptors involved were analyzed together with correlation with CAVD severity and risk factors.

**Results:**

Nucleotide and adenosine degradation rates were adversely modified on the aortic surface of stenotic valve as compared to ventricular side, including decreased ATP removal (1.25 ± 0.35 vs. 2.24 ± 0.61 nmol/min/cm^2^) and adenosine production (1.32 ± 0.12 vs. 2.49 ± 0.28 nmol/min/cm^2^) as well as increased adenosine deamination (1.28 ± 0.31 vs. 0.67 ± 0.11 nmol/min/cm^2^). The rates of nucleotide to adenosine conversions were lower, while adenosine deamination was higher on the aortic sides of stenotic vs. non-stenotic valve. There were no differences in extracellular nucleotide metabolism between aortic and ventricular sides of non-stenotic valves. Furthermore, nucleotide degradation rates, measured on aortic side in CAVD (*n* = 62), negatively correlated with echocardiographic and biochemical parameters of disease severity (aortic jet velocity vs. ATP hydrolysis: *r* = − 0.30, *p* < 0.05; vs. AMP hydrolysis: *r* = − 0.44, *p* < 0.001; valvular phosphate concentration vs. ATP hydrolysis: *r* = − 0.26, *p* < 0.05; vs. AMP hydrolysis: *r* = − 0.25, *p* = 0.05) while adenosine deamination showed positive correlation trend with valvular phosphate deposits (*r* = 0.23, *p* = 0.07). Nucleotide and adenosine conversion rates also correlated with CAVD risk factors, including hyperlipidemia (AMP hydrolysis vs. serum LDL cholesterol: *r* = − 0.28, *p* = 0.05; adenosine deamination vs. total cholesterol: *r* = 0.25, *p* = 0.05; LDL cholesterol: *r* = 0.28, *p* < 0.05; triglycerides: *r* = 0.32, *p* < 0.05), hypertension (adenosine deamination vs. systolic blood pressure: *r* = 0.28, *p* < 0.05) and thrombosis (ATP hydrolysis vs. prothrombin time: *r* = − 0.35, *p* < 0.01). Functional assays as well as histological and immunofluorescence, flow cytometry and RT-PCR studies identified all major ecto-enzymes engaged in nucleotide metabolism in aortic valves that included ecto-nucleotidases, alkaline phosphatase, and ecto-adenosine deaminase. We have shown that changes in nucleotide-converting ecto-enzymes were derived from their altered activities on valve cells and immune cell infiltrate. We have also demonstrated a presence of A1, A2a and A2b adenosine receptors with diminished expression of A2a and A2b in stenotic vs. non-stenotic valves. Finally, we revealed that augmenting adenosine effects by blocking adenosine deamination with deoxycoformycin decreased aortic valve thickness and reduced markers of calcification via adenosine-dependent pathways in a mouse model of CAVD.

**Conclusions:**

This work highlights profound changes in extracellular nucleotide and adenosine metabolism in CAVD. Altered extracellular nucleotide hydrolysis and degradation of adenosine in stenotic valves may affect purinergic responses to support a pro-stenotic milieu and valve calcification. This emphasizes a potential mechanism and target for prevention and therapy.

**Graphic abstract:**

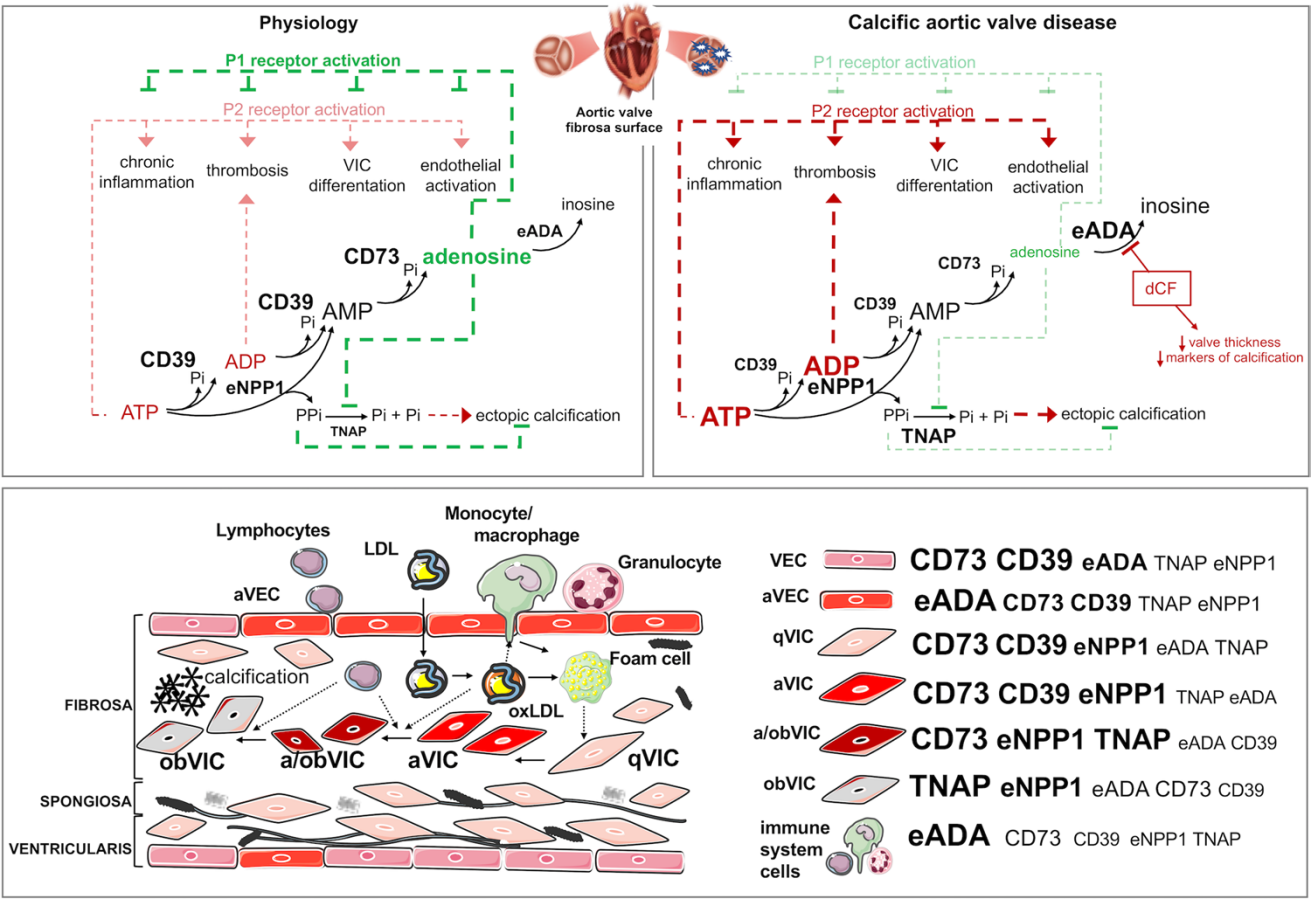
.

**Electronic supplementary material:**

The online version of this article (10.1007/s00392-019-01495-x) contains supplementary material, which is available to authorized users.

## Introduction

Calcific aortic valve disease (CAVD) is a slowly progressive disorder related to the mineralization of aortic valve leaflets [1]. Increased stiffness of the leaflets results in limited valve opening and leads to hemodynamic overload on the left ventricle, followed by valvular cardiomyopathy [2]. Currently, no medical therapies are available to prevent the development and progression of CAVD [3].

In early stages, CAVD is an active cell-regulated process initiated by endothelial disruption with macrophages and T cell infiltration with accumulation and oxidation of lipoproteins [4, 5]. These factors activate quiescent valvular interstitial cells (qVIC) to activated VICs (aVIC), which are characterized by the expression of smooth muscle cells alpha actin (α-SMA). Activation of VIC is associated with increased extracellular matrix production and remodeling as well as expression of matrix metalloproteinases and the secretion of proinflammatory cytokines, which all together result in pathological fibrosis and chronic inflammation of the valve [6, 7]. Simultaneously, in the presence of cytokines (TGF-β) and proteins associated with chondro- and osteogenesis, VICs undergo osteoblastic differentiation (obVIC), which is a direct cause of valve mineralization [8].

Another potent regulator of osteoblastic VIC differentiation is extracellular adenosine triphosphate (ATP), adenine nucleotide that acts by purinergic P2 receptors, and its breakdown product, adenosine that triggers cell-signaling effects by the activation of P1 receptors [9, 10]. In the cardiovascular system, ATP and ADP (adenosine diphosphate) are released by various cells, after their stimulation by shear stress, hypoxia, hyperglycemia, inflammation or platelets activators [11]. Despite the much lower concentration of ATP in extracellular space (nanomolar) than in cell (milimolar), its role as a signaling molecule seems to be important since it is known that nucleotides exist in the pericellular space at micromolar levels [12]. In addition to VIC differentiation, nucleotide receptor activation also affects such conditions as chronic inflammation by the stimulation of immune cell transmigration via endothelium or thromboregulation by platelets activation, which plays a significant role in CAVD initiation and progression [13–17].

Extracellular nucleotides are inactivated through the hydrolysis by cell-surface ecto-enzymes [18]. The first enzyme engaged in this cascade is an ecto-nucleoside triphosphate diphosphohydrolase 1 (CD39), which converts ATP to ADP and then to AMP (adenosine monophosphate) [19]. AMP is rapidly hydrolyzed by ecto-5′nucleotidase (CD73) to form adenosine, which is degraded by the last enzyme of this pathway, ecto-adenosine deaminase (eADA) that is fixed to the membrane by CD26 protein and/or adenosine receptors [20, 21]. Nucleotides may also be catabolized by other enzymes such as ecto-nucleotide pyrophosphatases/phosphodiesterases (eNPPs) or alkaline phosphatase (ALP) [22, 23]. Upstream pathways, which lead to extracellular ATP synthesis from AMP are the least important, because of the minimal ecto-kinase activity [24]. Except for the removal of nucleotides from the extracellular space, the significant function of ecto-nucleotidases is the production of adenosine, which attenuates inflammation and platelets reactivity [25–28]. Thus, the pericellular concentration of nucleotides and adenosine are strictly dependent on the production and breakdown of these molecules. Despite a few reports of selective changes in ecto-nucleotidase activities in CAVD, there is no overall assessment of extracellular nucleotide and adenosine metabolism pathways in the aortic valve.

Therefore, the aim of this study was to comprehensively examine extracellular nucleotide and adenosine metabolism in the human aortic valve in CAVD and to correlate the rates of valvular nucleotide conversions with CAVD severity and risk factors. For the first time, we have investigated the total flux between nucleotide degradation, adenosine production and its breakdown on both surfaces of aortic valves originated from control and CAVD patients. Additionally, to provide a potential molecular mechanism and a new target for early intervention of CAVD we have identified individual enzymes responsible for these changes, indicated their cellular origin and modulated their activity.

## Methods

An expanded methods section is available in the Supplementary material online.

### Patients and tissue collection

The study was performed based on the standards of the Declaration of Helsinki and it was approved by the local ethical committee. Informed consent has been obtained from the patients. Three leaflet aortic valves were collected during valve replacement for CAVD and named as stenotic aortic valves (total number of used valves: *n *= 71, mean age: 60, median age 62; 43 males; 28 females; age range: 36–74), while control aortic valves were obtained during heart transplantation or Bentall procedures and named as non-stenotic aortic valves (total number of used valves: *n *= 34, mean age: 53, median age: 53; 22 males; 13 females; age range: 28–75). The exact number of valves used for each experiment has been indicated in the figure legend. Dissected human aortic valve leaflets were immediately placed into ice-cold physiologic salt solution and transported to the laboratory on ice within 30 min of harvest.

### Determination of aortic valve surface ecto-enzymes activities

For the determination of ecto-enzymes activities, valve leaflets were weighed and washed in Hanks Balanced Salt Solution (HBSS). Then, aortic valve leaflets were divided into 0.2 cm^2^ sections and in this intact condition directly placed into incubation solution. The modified assay system based on exposition into incubation medium by fibrosa and ventricularis surfaces separately. An intact valve leaflet fragment was fixed under the 0.5 cm diameter hole drilled in the bottom of one well of 24-well plate. It was supported by a plastic plate and the pressure was adjusted to ensure an effective seal. The leaflet fragment, clamped between two plastic plates, fully sealed the area exposed to the incubation medium [29]. Next, each well has been washed twice with HBSS and 1 mL of HBBS with 50 µM adenosine, ATP or AMP was sequentially added with medium exchange after each substrate. 5 μm erythro-9-(2-hydroxy-3-nonyl)adenine (EHNA), an inhibitor of adenosine deaminase, was present during incubation with ATP and AMP to block the conversion of adenosine to inosine [30]. Although, nucleotides and nucleosides are maintained in extracellular space at nanomolar level, we adjusted the substrate concentration to micromolar as these compounds operate on the cell surface in the “pericellular halo” [21]. To ensure that evaluated activities originate exclusively from the action of extracellular enzymes, part of experiments were conducted with the nucleoside transport inhibitor: 5 μm S-(4-Nitrobenzyl)-6-thioinosine (NBTI) [31]. After 0, 5, 15 and 30 min of incubation at 37 °C samples were collected and concentrations of nucleotides and nucleosides were measured by reversed-phase HPLC according to the method described earlier [32]. Enzyme activities were calculated from linear phase of the reaction and in the main experiment, the rates were normalized to the surface area. Final results for each patient based on the average activity obtained from 3 valve leaflets. After the experiment, valve leaflet fragments were washed in HBSS, dried and frozen at − 80 °C for later use.

### Determination of valve deposits compounds concentrations

Sections of aortic valve leaflets, previously used for the estimation of ecto-enzymes activities, were quickly thawed and dissolved separately in 6 m HCl at 95 °C for 24 h followed by centrifugation at 2000×*g* during 30 min. The supernatant was collected and diluted with deionized water and used for the determination of calcium and magnesium or diluted with 0.6 m H_2_SO_4_ for phosphate determination as described in Supplementary material online.

### Pre-operative echocardiography, blood pressure measurement and biochemical blood analyzes

All patients underwent a Doppler pre-operative echocardiographic examinations of aortic jet velocity (*V*_max_) and mean transvalvular gradient using the Vivid Q Portable Ultrasound (GE Healthcare, USA). Blood pressure measurement was taken with a validated electronic device, and it was the mean of two readings. For biochemical analysis, blood samples were collected from all subjects in a fasting state and serum or citrate plasma were obtained by centrifugation (1700×*g*, 10 min, 21 °C). Parameters of the coagulation system (prothrombin time, international normalized ratio), lipid profile and carbohydrate metabolism parameters (glycemia and glycated hemoglobin HbA1c) were measured in patients using standard methods.

### Animal studies

All experiments were conducted in accordance with the guide for the care and use of laboratory animals published by the European Parliament, Directive 2010/63/EU and were approved by the local Bioethical Committee.

Male C57BL/6 J mice (wild type, WT) and double knock-out for apolipoprotein E (ApoE) and low-density lipoprotein receptor (LDLR) on the C57BL/6 J background (ApoE−/−LDLR−/−) [33], originally obtained from Jackson Lab (USA) were bred in house and used for the experiments at the age of 3, 6 or 10 months. Animals had an unlimited access to water and standard chow diet. Blood samples were collected by tail vein puncture. Serum was obtained after centrifugation (1700×*g*, 10 min, 21 °C). Mice were sacrificed under anaesthesia by a intraperitoneal injection of ketamine and xylazine (100 mg/kg/10 mg/kg). Isolated fragments of aortic roots were cleaved from a perivascular adventitia in cold HBSS and used for the measurements of nucleotides and adenosine conversion rates. For histological and immunofluorescence analyses, aortic roots were collected, placed into an Optimal Cutting Temperature (OCT) compound, and snap-frozen at − 80 °C or preserved in 4% buffered formalin solution and embedded in paraffin.

### Treatment of ApoE−/−LDLR−/− mice

4-month-old ApoE−/−LDLR−/− mice were treated with adenosine deaminase inhibitor, deoxycoformycin (pentostatin, dCF) as shown before [29]. Saline-treated ApoE−/−LDLR−/− mice were used as controls. Isolated fragments of aortic roots were cleaned as described above and used for the measurement of ALP activity. For histological analysis of aortic valve thickness, aortic roots were placed into an OCT compound and snap-frozen at − 80 °C. Serum samples were obtained as described above and used for the measurement of ALP activity, calcium, magnesium and phosphate concentration by standard methods using automated chemistry analyzer XL180 analyzer (ERBA Mannheim, Germany) Biochemica. The efficiency of dCF in blocking ADA and the effects of dCF in this animal model on endothelial function, blood nucleotide and adenosine concentration, biochemical parameters and morphology were investigated earlier [29].

### Histological analysis

Representative non-stenotic and stenotic aortic valve leaflets or mice aortic roots were fixed in 4% buffered formaldehyde, decalcified (if necessary) and embedded in paraffin. Then, the paraffin-embedded aortic valve leaflets or aortic roots were cut into 5 μm-thick cross-sections using a histological microtome, placed on microscopic slides and deparaffinized prior to staining. Aortic valve sections were stained with hematoxylin and eosin (HE) for general morphology. For the assessment of specific aortic valve morphology, adjacent sections were stained according to Masson’s Trichrome (TR) standard protocol and Orcein Martius Scarlet Blue (OMSB) protocol [34]. These stainings allowed to characterize non-stenotic and stenotic valve composition, including cellular components as well as extracellular matrix fibers (loose connective tissue), collagen fibers (dense connective tissue), calcium nodules and myofibroblast-like cells, which far exceeds the capabilities of standard staining for calcium deposits. Mice aortic roots were stained according to OMSB and Oil Red O protocols [34, 35]. Human valvular interstitial cells were stained on 24-well-plate using von Kossa staining protocol [36]. The acquisition and processing of stained section images were described in the Supplementary material online.

### Immunofluorescence analysis

Adjacent aortic valve sections to sections used for histological stainings were used to immunofluorescence analysis (IF). 5 μm-thick paraffin-embedded aortic valve cross-sections were collected on polylysine-covered microscopic slides and deparaffinized using a standard protocol. Next, sections were pretreated according to the citrate-base HIER (Sigma) protocol to unmask the antigens and epitopes in formalin-fixed and paraffin-embedded sections. The OCT-frozen mice aortic roots originated from 6-month-old male WT and ApoE−/−LDLR mice were cut into 10 μm-thick cross sections (using Leica CM1920 cryotome), using a standardized protocol. Aortic root cross-sections were collected on polilisine-covered microscopic slides and fixed in acetone for 10 min. Human primary aortic valve endothelial (hVEC) and interstitial (hVIC) cells intended to IF were seeded on 96-well optical-bottom plate (Nunc ThermoFisher, USA) at a density 1 × 10^4^ cells/well in a total volume of 200 μL cell culture medium. 24 h after seeding of hVEC and 72 h after seeding of hVIC, cell culture medium was removed and rinsed 3 times with PBS. Cells were fixed using 100 μL 4% paraformaldehyde (pH 7.4) for 10 min at 37 °C. Paraformaldehyde was removed and washed three times with PBS. To reduce non-specific antibody binding, slices or cells were preincubated with a PAD solution (5% of normal goat serum and 2% of filtered dry milk) (Sigma). The antibodies used and their origin were described in the Supplementary material online. Primary antibodies were used at 1:100 final dilution (1 h incubation), while secondary antibodies at 1:600 (30 min incubation). Negative controls omitted the primary antibodies (data not shown). Cell nuclei were counterstained with Hoechst 33258 (Sigma) (1:1500 final dilution, 5 min incubation). Images were recorded with an AxioCam MRc5 camera and an AxioObserved.D1 inverted fluorescent microscope (Zeiss) with appropriate filter cubes to show Cy3 (red), Alexa Fluor 488 (green) and Hoechst 33258 (blue) fluorescence, stored as tiff files and analyzed automatically using the Columbus Image Data Storage and Analysis System (Perkin Elmer). Total human CD39, CD73, eNPP1, ALP, ADA, A1R, A2aR, A2bR and A3R positive area in aortic valves were measured in each slide and the percentage of total aortic valve cross-sectional area covered by red signal was calculated from six sections.

### Gene expression

Human non-stenotic and stenotic aortic valves were directly lysed with QIAzol^®^ Lysis Reagent (Qiagen, Hilden, Germany) by shaking (5 min) in the presence of 3 mm diameter solid glass beads (Sigma, USA). Total RNA was isolated with RNeasy mini kit (Qiagen) according to the manufacturer’s instruction. To prevent DNA contamination, samples were pretreated with RNase-free DNase (Qiagen). The concentration of RNA was calculated based on the absorbance at 260 nm. RNA samples were stored at − 70 °C until use. For the measurement of CD39, CD73, ADA, ADORA1, ADORA2a, ADORA2b and ADORA3 mRNA expression, TaqManOne-Step RT-PCR Master MixReagents (Applied Biosystems, USA) were used as described previously [37, 38] according to the manufacturer’s protocol. The relative expressions were calculated using the comparative relative standard curve method [39]. We used housekeeping gene, TATA-binding protein (TBP), as the relative control. TaqMan probes ids were given in the Supplementary material online.

### Non-stenotic aortic valve cells isolation and culture

Aortic valve endothelial (hVEC) and interstitial (hVIC) cells were isolated from non-stenotic human aortic valves as was shown in Fig. 4a. The valve was digested with 5 mL collagenase A (0.15% *w*/*v*) for 10 min at 37 °C to obtain hVEC. 5 mL of EBM-2 Medium (Lonza, USA) was added to stop the action of collagenase. To isolate hVIC, the valve was minced and further digested with 5 mL collagenase A (0.15% *w*/*v*) for additional 45 min at 37 °C. 5 mL of DMEM (Sigma, USA) supplemented with 1 mmol/L l-glutamine, 10% FBS and 1% penicillin/streptomycin (*v*/*v*) (Sigma, USA) was added to neutralize the collagenase. Each of the suspensions, hVEC and hVIC, was purified using mesh filters 100 μm, 70 μm, 40 μm and centrifuged (150×*g*, 4 min). After centrifugation, hVEC pellet was resuspended in EBM-2 Medium (Lonza, USA), while hVIC pellet in a standard Dulbecco’s Modified Eagle’s medium (DMEM, Sigma, USA) supplemented with 1 mmol/L l-glutamine, 10% FBS and 1% penicillin/streptomycin (*v*/*v*) (Sigma, USA). Cells were cultured at 37 °C, in 5% CO_2_ atmosphere and used for experiments at passage 4.

### Stenotic aortic valve cells isolation

Aortic valve endothelial and interstitial cells, as well as immune infiltrate, were also isolated from stenotic human aortic valves as was shown in Fig. S4a. hVEC and immune cells located in the upper layers of the valve were isolated after 10 min incubation with agitation in 5 mL of collagenase A (0.15% *w*/*v*) at 37 °C. 5 mL of EBM-2 Medium (Lonza, USA) was added to stop the action of collagenase. Aortic valve transport medium and suspension obtained after the first step of isolation were purified using mesh filters 100 μm, 70 μm, 40 μm. After centrifugation (150x*g*, 4 min), pellets were resuspended in MACS buffer, pooled and used for FACS analysis. hVIC and immune cells derived from the deeper layers of the valve were isolated after mincing of the valve and additional digestion for 45 min in 5 mL of collagenase A (0.15% *w*/*v*) at 37 °C. 5 mL of DMEM (Sigma, USA) supplemented with 1 mmol/L l-glutamine, 10% FBS and 1% penicillin/streptomycin (*v*/*v*) (Sigma, USA) was added to neutralize the collagenase. Petri place used for mincing of the tissue was washed using PBS and collected solution and suspension obtained after the second step of isolation were purified using mesh filters 100 μm, 70 μm, 40 μm. After centrifugation (150x*g*, 4 min), pellets were resuspended in MACS buffer, pooled and used for FACS analysis.

### Peripheral blood mononuclear cells isolation

Peripheral blood mononuclear cells (PBMC) were isolated from healthy adult donors using a Histopaque procedure as described in the Supplementary material online.

### Determination of specific ecto-enzyme activities on the surface of human aortic valve cells, immune cells and mice aortic roots

hVEC and hVIC isolated from non-stenotic aortic valves were used at passage 4 and seeded on 24-well plates at a density of 0.05 × 10^6^ cells/well. hVIC treated with osteogenic medium was cultured for 19 days in cell culture medium supplemented with 3 mm phosphate with medium exchange every 2 days. Cells were used for the experiments at 90–100% confluency and washed with HBSS. Isolated human peripheral blood mononuclear cells and monocyte/macrophage cells (SC line, ATCC, cat. CRL-9855) that were used at passage 4, were plated in 24-well cell culture plate at a density 0.2 × 10^6^ per well in a total volume of 1 mL HBSS. Mice aortic roots were cleaned of surrounding tissues as described above and used for experiment. Cells or mice aortic roots were pre-incubated in HBSS for 15 min at 37 °C with specific ecto-enzyme inhibitors, including 5 μm erythro-9-(2-hydroxy-3-nonyl) adenine for ADA1, 150 μm adenosine 5′-(α,β-methylene)diphosphate (AOPCP) for CD73 [40], 500 μm levamisole hydrochloride for ALP [41], 150 μm 6-*N*,*N*-Diethyl-β-γ-dibromomethylene-d-adenosine-5′-triphosphate trisodium salt hydrate (ARL67156) for ecto-ATPases, mainly NTPDases (including CD39) [42, 43] and 50 μm pyridoxal phosphate-6-azo(benzene-2,4-disulfonic acid) tetrasodium salt hydrate (PPADS) for ENPPs [44]. After pre-incubation ecto-enzyme substrates were added (50 μm adenosine, AMP or ATP) and cells were incubated at 37 °C for 30 min. Samples of the incubation medium were collected in 0, 5, 15 and 30 min time points and analyzed for the concentration of nucleotides and their catabolites using HPLC as described above. Enzyme activities were calculated from linear phase of the reaction and normalized per cell protein concentration or aortic root surface area.

### Determination of alkaline phosphatase activity on the surface of mice aortic roots

ALP activity on aortic roots obtained from ApoE−/−LDLR−/− mice treated in vivo with dCF and from WT mice that were treated ex vivo with osteogenic medium (DMEM supplemented with 1 mmol/L l-glutamine, 10% FBS and 1% penicillin/streptomycin (*v*/*v*) and 3 mm phosphate), adenosine (50 μm) and adenosine receptor antagonists (50 μm) in the presence of 150 μm AOPCP, 5 μm dCF and 5 μm NBTI has been measured as described in the Supplementary material online.

### Flow cytometry analysis

Cells were resuspended in MACS buffer, preincubated with FcR Blocking Reagent (Miltenyi Biotech) and stained with specific antibodies that origin was described in the Supplementary material online.

To identify aortic valve endothelial cells and individual subsets of aortic valve interstitial cells and immune cells we used a panel of antibodies against different cell-specific markers, including markers for endothelial cells (CD45−, CD31^high^), activated VIC (CD45−, Vim+, Sial−, αSMA^high^), activated/osteoblast-like VIC (CD45−, Vim+, Sial+, αSMA^int^), osteoblast-like VIC (CD45−, Vim+, Sial+, αSMA−), T helper cells (CD45+, CD8+), T cytotoxic cells (CD45+,CD4+), B cells (CD45+, CD19+), monocytes/macrophages (CD45+, CD11b+, CD14+) and granulocytes (CD45+, CD11b^int^, CD14−). After 5 min of the incubation at room temperature cells were washed and resuspended in 200 µL MACS buffer for flow cytometry. Cell measurements were performed and analyzed as described in the Supplementary material online. The different cells subsets were enumerated and the percentage of CD39, CD73 and CD26 (adenosine deaminase binding-protein) and corresponding expression levels as measured by mean fluorescence intensity (MFI) was assessed.

### Statistical analysis

Statistical analysis was performed using InStat software (GraphPad, San Diego, CA). Comparisons of mean values between groups were evaluated by one-way analysis of variance (ANOVA) followed by Holm–Sidak, or Sidak post hoc tests, two-way ANOVA followed by Sidak post hoc test, unpaired Student’s *t* test, or Mann–Whitney *U* test, as appropriate. Normality was assessed using the Kolmogorov–Smirnov test (when *n* = 5), Shapiro–Wilk test (when *n* = 7), and the D’Agostino and Pearson Omnibus (when *n* ≥ 8) normality tests. The exact value of n was provided for each type of experiments. Statistical significance was assumed at *p *< 0.05. Error bars indicated the standard error of the mean (SEM) unless otherwise described in the figure legend.

## Results

### Changes in nucleotide and adenosine degradation rates on the surface of intact aortic valves correlate with CAVD severity

**Fig. 1:**
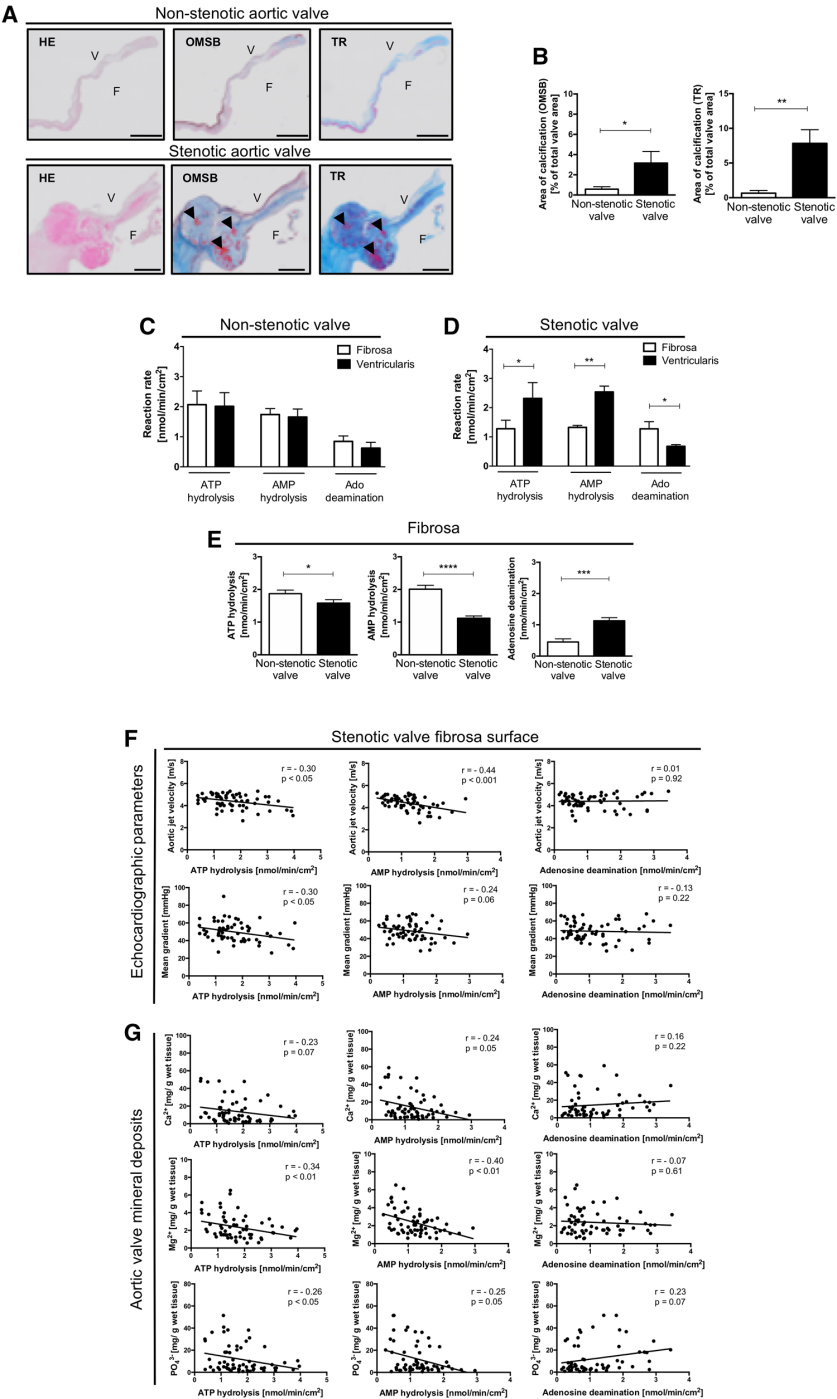
Activities of nucleotide metabolism ecto-enzymes are shifted towards nucleotide accumulation and adenosine degradation on the fibrosa surface of stenotic aortic valve and correlated with CAVD severity parameters. Representative images of non-stenotic and stenotic aortic valves stained with Hematoxilin and Eosin (HE). Orcein Mertius Scarlet Blue (OMSB) and Masson’s Trichrome (TR) *F* fibrosa, *V* ventricularis. Scale bar = 2 mm (**a**). HE staining was used for general microscopy. In OMSB staining cell nuclei were stain red, while purple/grey sections represent elastic fibers and elastic laminae, blue sections represent collagen fibers and red nodules represent calcium nodules. In TR staining, cell nuclei were stain dark pink/red, dark blue sections represent collagen fibers (dense connective tissue), light blue sections represent extracellular matrix fibers (loose connective tissue), purple nodules represent calcium nodules and red fibers represent myofibroblast-like cells. Calcium nodules were pointed by black arrows. Quantitative analysis of aortic valve calcification area (**b**) in non-stenotic (*n *= 4) and stenotic (*n *= 3) aortic valves. Areas of calcification were assessed in six cross-sections per each valve. Results are shown as mean ± SEM; *p < 0.05; **p < 0.01; ***p < 0.001; ****p < 0.0001 vs. non-stenotic valve by Mann–Whitney test. Rates of ATP hydrolysis, AMP hydrolysis and adenosine deamination of non-stenotic (**c**) and stenotic (**d**) aortic valves. Results are shown as mean ± SEM, *n *= 15–18 aortic valve leaflets; *p < 0.01, **p < 0.01 vs. fibrosa by two-way ANOVA followed by Sidak post hoc test. Rates of ATP hydrolysis, AMP hydrolysis and adenosine deamination (**e**) on the fibrosa surface of non-stenotic (*n *= 24) and stenotic (*n *= 62) aortic valves. The average rate of nucleotide or adenosine convertion for each valve was estimated from measurements for three leaflets independently, in the sites free of calcification. Results are shown as mean ± SEM; **p < 0.01; ***p < 0.001; ****p < 0.0001 vs. non-stenotic valve by Mann–Whitney test. The correlation analysis of ecto-enzyme activities estimated on the fibrosa surface of stenotic aortic valves in sections free of massive calcifications and valve deposit concentration measured in these sections and pre-operative echocardiography parameters (**f**) and valvular calcium, magnesium and phosphate deposit concentration (**g**) in the study group of patients with CAVD (*n *= 62). Results are shown as Spearman correlation coefficient and* p* value

Nucleotide and adenosine degradation rates were determined on the surface of intact non-stenotic and stenotic aortic valves using three free of calcification sections, each from a single leaflet. Representative images of histological stainings of analyzed valves revealed altered structure of stenotic valves that showed the presence of calcification (red nodules in OMSB staining and purple nodules in TR staining) and a change in the composition of extracellular matrix (stenotic valves represented more dense connective tissue, which stains dark blue in TR and showed myofibroblast-like cells as red fibers in TR) (Fig. 1a, b).

First, total activities of adenine nucleotide catabolism ecto-enzymes in intact non-stenotic (Fig. S1a–c) and stenotic (Fig. S1d–f) aortic valves were analyzed with two different assay methods. The use of a first method (Fig. S1a, d), which assumed the incubation of entire, free of calcifications, leaflet section in the substrate solution, resulted in a higher ATP hydrolysis, AMP hydrolysis and adenosine deamination than exposition of only one side (aortic side, fibrosa) of the aortic valve leaflet (method 2, Fig. S1b, e). Since the second method allowed for the estimation of these activities on the fibrosa and ventricularis surfaces separately, it has been used in further experiments. Nucleotide and adenosine degradation rates did not differ between fibrosa and ventricularis of non-stenotic aortic valves (Fig. 1c). In contrary, in stenotic aortic valves, the rates of ATP and AMP hydrolysis were lower, while adenosine deamination was higher on the fibrosa than on ventricularis surface (Fig. 1d).

Next, we determined whether the rates of nucleotide degradation fluctuate between different leaflets of the same aortic valve. Analyzing three different sections of each leaflet, we exhibited that the rate of extracellular nucleotide and adenosine metabolism did not differ between leaflets within the same non-stenotic (Fig. S1g) and stenotic (Fig. S1h) aortic valve.

Comparing nucleotide and adenosine degradation rates on the fibrosa surface in a representative study group (*n *= 86) that were characterized in Table 1, we observed lower ATP and AMP hydrolysis, as well as higher adenosine deamination, in stenotic aortic valve than in non-stenotic (Fig. 1e). The blockade of a transmembrane nucleoside transport by NBTI did not affect the rate of product formation that indicates the contribution of only extracellular enzymes in these changes (Fig. S2).

**Table 1:** Patient characteristics

	Control	Aortic stenosis	*p* value
Age, years	53 ± 3	60 ± 2	0.06
Female/male	6/18 (25/75%)	26/36 (42/58%)	–
Body weight (kg)	80 ± 3.0	81 ± 2.0	0.79
Echocardiographic parameters			
Aortic jet velocity (m/s)	2.05 ± 0.10	4.43 ± 0.08	< 0.0001
Mean transvalvular gradient (mmHg)	16.5 ± 1.21	48.2 ± 1.45	< 0.0001
Aortic valve deposits			
Calcium (mg/g wet tissue)	4.6 ± 0.5	17 ± 2.4	0.002
Magnesium (mg/g wet tissue)	0.8 ± 0.2	2.5 ± 0.2	< 0.0001
Phosphate (mg/g wet tissue)	2.3 ± 0.8	12.5 ± 1.8	0.0008
Lipid profile			
Total cholesterol (mg/dL)	162 ± 7.9	187 ± 6.7	0.04
Low density lipoproteins (mg/dL)	96 ± 12.0	113 ± 5.8	0.16
Triglycerides (mg/dL)	117 ± 7.9	126 ± 7.6	0.50
High density lipoproteins (mg/dL)	43 ± 3.1	49.3 ± 1.8	0.07
Coagulation parameters			
Prothrombin time (s)	12.2 ± 0.31	11.9 ± 0.13	0.29
International normalized ratio	1.05 ± 0.02	1.04 ± 0.01	0.62
Blood pressure			
Systolic pressure (mm Hg)	129 ± 6.3	131 ± 2.3	0.14
Diastolic pressure (mm Hg)	73 ± 2.2	75 ± 1.4	0.45
Glycemia			
Fasting glucose (mg/dL)	105 ± 4.7	113 ± 4.8	0.34
Glycated hemoglobin HbA1c (%)	5.60 ± 0.08	6.16 ± 0.14	0.02
Comorbidities			
Aortic regurgitation	0 (0%)	62 (100%)	–
Aortic insufficiency	20 (83%)	21 (34%)	–
Aortic aneurysm	13 (54%)	17 (27%)	–
Hypertension	11 (46%)	40 (65%)	–
Coronary artery disease	4 (16%)	23 (37%)	–
Hyperlipidemia	6 (24%)	32 (51%)	–
Diabetes mellitus	2 (8%)	19 (31%)	–
Pharmacotherapy			
Antihypertensives			
Calcium channel blockers	3 (13%)	13 (21%)	–
ACE inhibitors	4 (16%)	18 (29%)	–
Angiotensin receptor antagonists	2 (8%)	10 (16%)	–
Adrenergic receptor antagonists	6 (33%)	41 (66%)	–
Diuretics	3 (13%)	29 (47%)	
Statins	4 (15%)	31 (50%)	–
Antithrombotics			
Antiplatelet drugs	9 (38%)	30 (48%)	–
Anticoagulants	2 (8%)	4 (6%)	–
Antidiabetics			
Insulin	0 (0%)	6 (10%)	–
Biguanides	1 (4%)	8 (13%)	–
Sulfonylureas	1 (4%)	4 (6%)	–

**Fig. 2:**
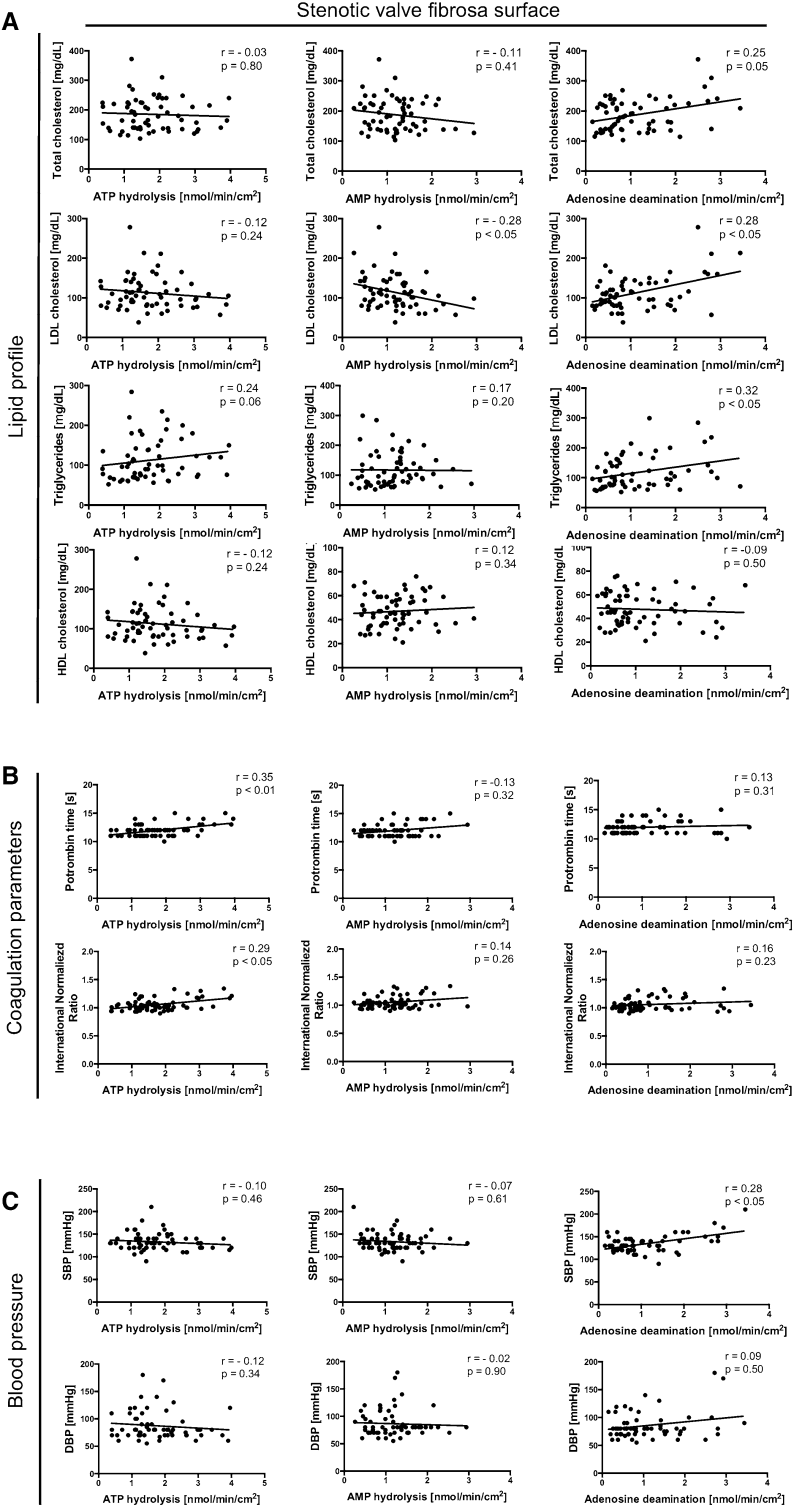
Changes in activities of nucleotide metabolism ecto-enzymes measured on the fibrosa surface of stenotic valve correlate with CAVD risk factors. The correlation analysis of ecto-enzyme activities estimated on the fibrosa surface of stenotic aortic valves in sections free of massive calcifications and serum lipid profile parameters (**a**), coagulation parameters (**b**) and blood pressure (**c**) in the study group of patients with CAVD (*n *= 62). Results are shown as Spearman correlation coefficient and *p* value

Clinical characteristics of control patients (*n *= 24) and aortic valve stenosis patients (*n *= 62) included for the analysis of nucleotide and adenosine degradation rates on the fibrosa surface of the valve (Fig. 2). Results are shown as mean ± SEM or percentage

The correlation analysis of ecto-enzyme activities with pre-operative echocardiographic parameters of aortic stenosis showed negative correlation of the rates of ATP and AMP hydrolysis measured on the fibrosa surface of sections free of calcifications with aortic jet velocity and mean gradient across aortic valve (Fig. 1f). ATP and AMP hydrolysis also negatively correlated with the concentration of calcium, magnesium and phosphate deposits in stenotic aortic valves, while the rate of adenosine degradation tended to negative correlation with calcium and phosphate deposits (Fig. 1g). Moreover, nucleotide and adenosine degradation rates measured on the fibrosa surface of stenotic valve correlated with serum lipid profile, coagulation parameters and blood pressure in the study group. AMP hydrolysis negatively correlated with serum LDL cholesterol, while adenosine deamination correlated positively with serum total cholesterol, LDL cholesterol and triglycerides (Fig. 2a). In turn, ATP hydrolysis positively correlated with prothrombin time and international normalized ratio (Fig. 2b), while adenosine deamination positively correlated with systolic blood pressure (Fig. 2c). There were no significant correlations of nucleotide and adenosine convertion rates with glucose metabolism parameters (data not shown).

### The presence of specific extracellular nucleotide and adenosine metabolism enzymes in aortic valves

**Fig. 3:**
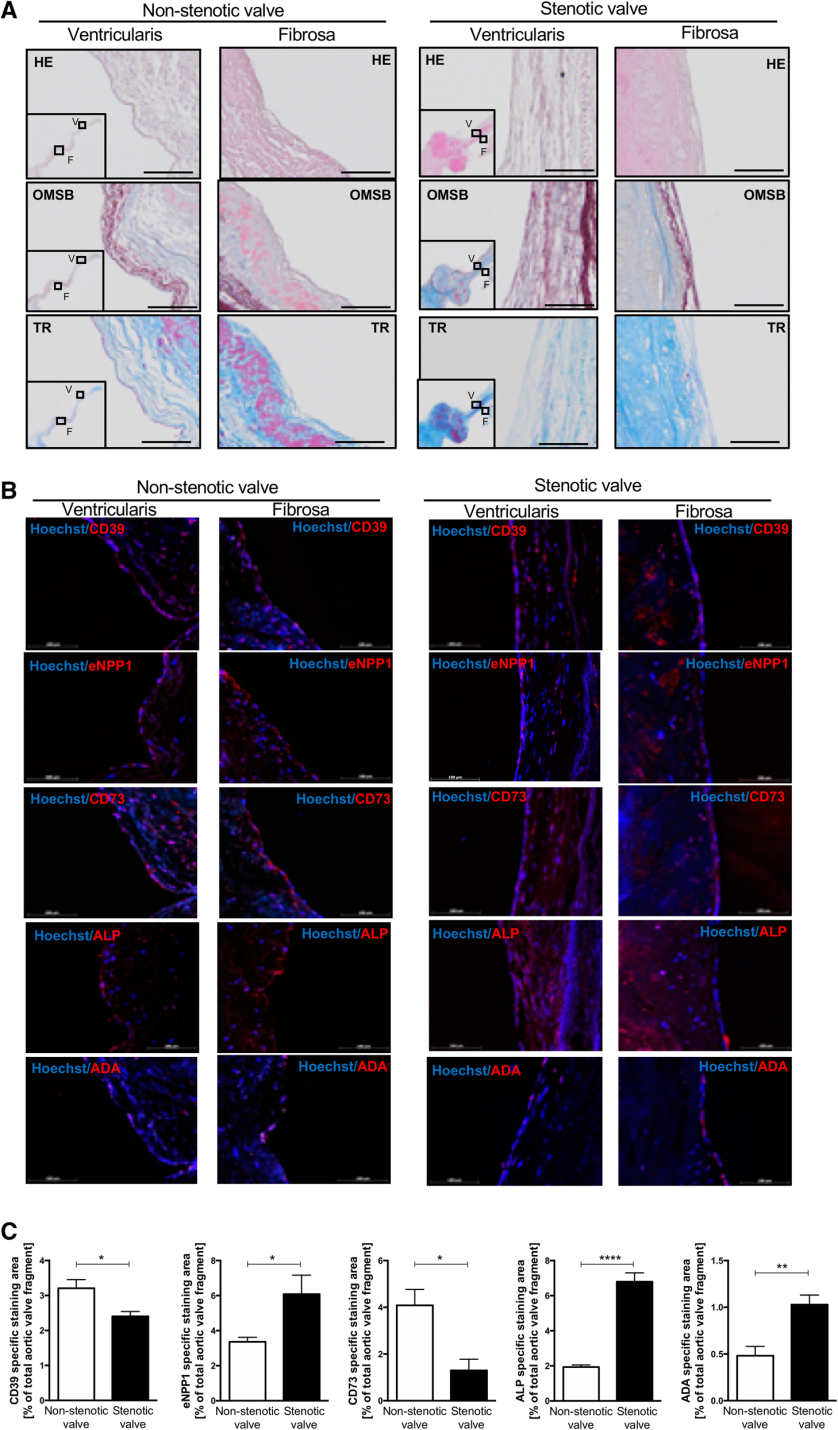
Human aortic valves, both non-stenotic and stenotic express nucleotide metabolism ecto-enzymes including ecto-nucleoside triphosphate diphosphohydrolase 1, ecto-nucleotide pyrophosphatase/phosphodiesterase 1, ecto5′nucleotidase, alkaline phosphatase and adenosine deaminase. Representative images of fibrosa and ventriculatis of analyzed aortic valves stained with Hematoxilin and Eosin (HE), Orcein Mertius Scarlet Blue (OMSB) and Masson’s Trichrome (TR) (**a**). Scale bar = 100 μm. Representative images of matching sections stained by immunofluorescence (red signal) for CD39 (ecto-nucleoside triphosphate diphosphohydrolase 1), eNPP1 (ecto-nucleotide pyrophosphatase/phosphodiesterase 1), CD73 (ecto5′-nucleotidase), ALP (alkaline phosphatase) and ADA (adenosine deaminase) (**b**). Quantitative analysis of CD39, eNPP1, CD73, ALP and ADA positive area (**c**) that corresponds to the specific signal for each enzyme for non-stenotic (*n* = 3) and stenotic (*n* = 4) aortic valves was assessed in six cross-sections per each valve. Fluorescence values of the negative control slices were substracted from the fluorescence value of the stained slices. Results are shown as mean ± SEM

At the next stage, we analyzed which enzymes involved in extracellular nucleotide and adenosine metabolism are found in aortic valves. For this purpose, microphotographs of histological stainings for representative aortic valves (Fig. 3a) had been complied with immunofluorescence analysis (Fig. 3b). These results indicated that in both stenotic and non-stenotic aortic valves are enzymes that can be engaged in nucleotide and adenosine metabolism, including ecto-nucleoside triphosphate diphosphohydrolase 1 (eNTPD1, CD39), ecto-nucleotide pyrophosphatase/phosphodiesterase 1 (eNPP1), ecto-5′-nucleotidase (e5NT, CD73), alkaline phosphatase (ALP) and adenosine deaminase (ADA). Using the same fluorescence microscope settings, we determined the area of a specific signal for each enzyme (Fig. 3c). In a non-stenotic aortic valve, the most abundant signal area was observed for CD73, CD39, and eNPP1, while in stenotic valve the highest signal area was observed for ALP and eNPP1 with a diminished signal for CD73 and CD39. Signal area for ADA was minor in both types of the valve, but it was directed towards a larger area in the stenotic valve. Since, increased expression of eNPP1 and ALP in stenotic aortic valves have been previously described [22, 45], in this study we focused on CD73, CD39 and ADA mRNA expressions that were measured in aortic valve sections free of calcifications. The expressions of CD73 and CD39 were lower in stenotic aortic valves than in non-stenotic (Fig. S3a), while no changes in ADA mRNA were observed. As it has been reported that the expression of CD73 may occur at the sites of calcification [10, 46], we analyzed the localization of nucleotide metabolism enzymes in the areas of calcification by immunofluorescence and observed the accumulation of the signal for CD73 and ALP within these sites (Fig. S3b, c).

### The activity of specific extracellular nucleotide and adenosine metabolism enzymes on non-stenotic aortic valve cells

**Fig. 4:**
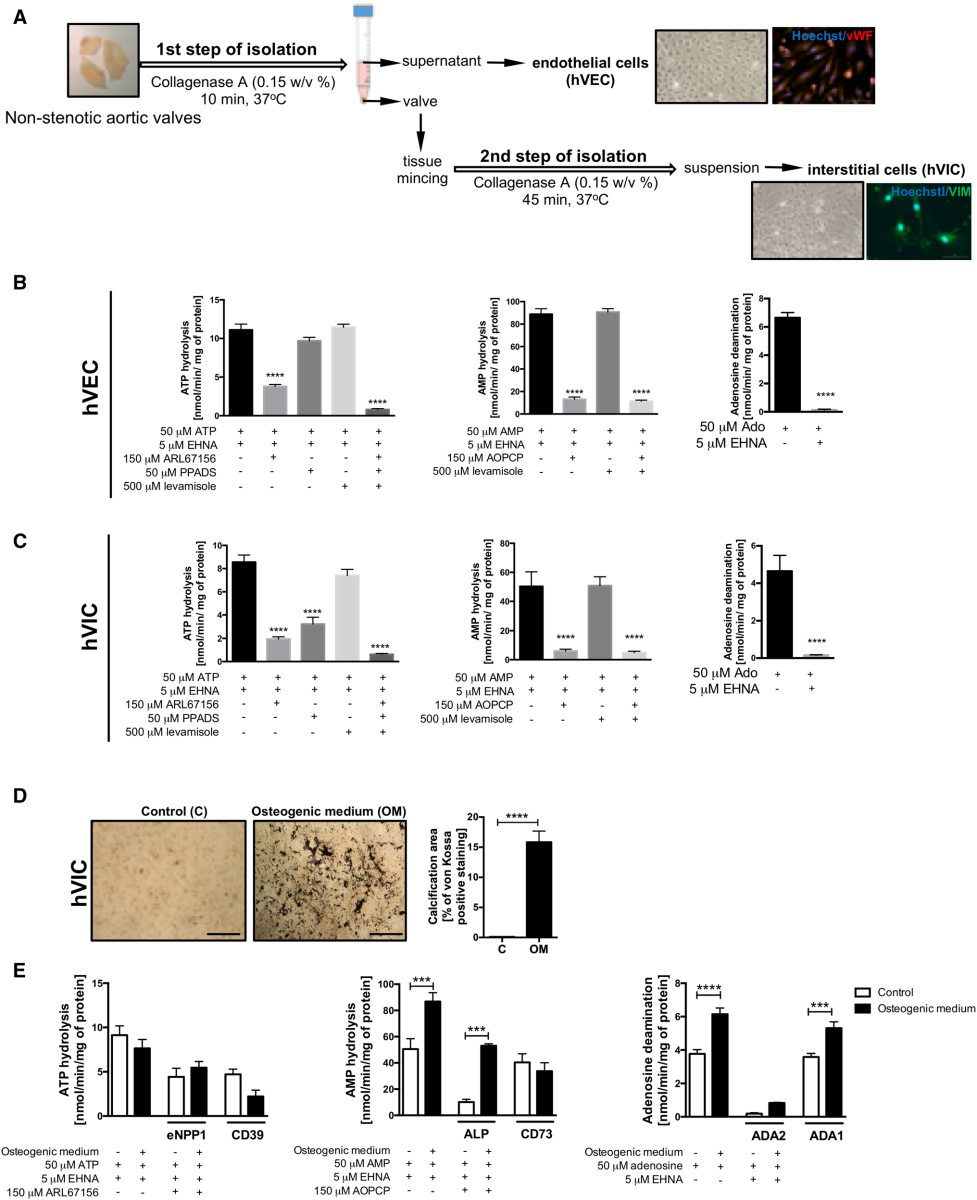
Aortic valve endothelial and interstitial cells are the main source of nucleotide-degrading ecto-nucleotidases. Simplified protocol of non-stenotic aortic valve cell isolation, including endothelial cells (first step of isolation, vWF positive) and interstitial cells (second step of isolation; Vimentin positive, Vim+) (**a**). The rates of ATP hydrolysis, AMP hydrolysis and adenosine deamination on the surface of human aortic valve endothelial cells (hVEC; **b**) and interstitial cells (hVIC; **c**) in the presence of specific ecto-enzyme inhibitors. Results are shown as mean ± SEM; *n *= 9 (independent isolations from 3 patients), *p < 0.05, ***p < 0.001, ****p < 0.0001 vs. without inhibitors or control staining by one-way Anova followed by Holm–Sidak post hoc test or student *t* test. von Kossa staining of calcium deposits (black nodules) in hVIC that were cultured for 19 days with control or osteogenic medium (with 3 mm phosphate) and calcification area in hVIC culture presented as a percentage of von Kossa positive staining (**d**). The rates of ATP hydrolysis, AMP hydrolysis and adenosine deamination on the surface of hVIC cultured in control and osteogenic medium in the presence of specific ecto-enzyme inhibitors (**e**). The activities of particular ecto-enzymes were estimated or calculated after the incubation with these inhibitors. Results are shown as mean ± SEM; *n *= 6, ***p < 0.001, ****p < 0.0001 vs. control cells by one-way Anova followed by Holm–Sidak post hoc test or student *t* test

To compare the results obtained from previous analyzes using aortic valves, then we investigated nucleotide and adenosine metabolism on the surface of aortic valve cells. Aortic valve endothelial and interstitial cells isolated from human non-stenotic aortic valves (Fig. 4a) actively degraded nucleotides and adenosine on their surface (Fig. 4b, c). Using ecto-enzyme inhibitors, we observed that after incubation with ARL67156, about 70% of ATP hydrolysis was inhibited on both hVEC (Fig. 4b) and hVIC (Fig. 4c). After the incubation with PPADS, we observed only about 10% inhibition of ATP hydrolysis on hVEC (Fig. 4b) and about 60% of inhibition on hVIC (Fig. 4c). This indicates a key role of CD39 in ATP hydrolysis on hVEC and both ecto-nucleotidases (CD39 and eNPP1) on hVIC. Levamisole (ALP inhibitor) did not affect the ATP hydrolysis on both cell types (Fig. 4b, c) The rate of AMP hydrolysis was decreased after the addition of AOPCP about 80% on both, hVEC (Fig. 4b) and hVIC (Fig. 4c), while levamisole did not affect AMP hydrolysis on both types of cells (Fig. 4b, c). EHNA abolished more than 90% of extracellular adenosine deamination on hVEC (Fig. 4b) and hVIC (Fig. 4c). Then we analyzed ecto-enzyme activities on hVIC isolated from human non-stenotic aortic valves that were cultured in high phosphate osteogenic medium. 19 days culture in osteogenic medium induced mineralization of VIC (Fig. 4d) with no significant changes in extracellular ATP hydrolysis, highly increased AMP hydrolysis that originated from heightened ALP activity and increased adenosine deamination that was derived from ADA1 activity (Fig. 4e). Based on obtained results we calculated the remained activities that reflected CD39 and CD73 and observed a tendency to lower rates of CD39-dependent ATP hydrolysis and CD73-dependent AMP hydrolysis (Fig. 4e).

### The origin of individual enzymes of extracellular nucleotide and adenosine metabolism in stenotic aortic valve cells

**Fig. 5:**
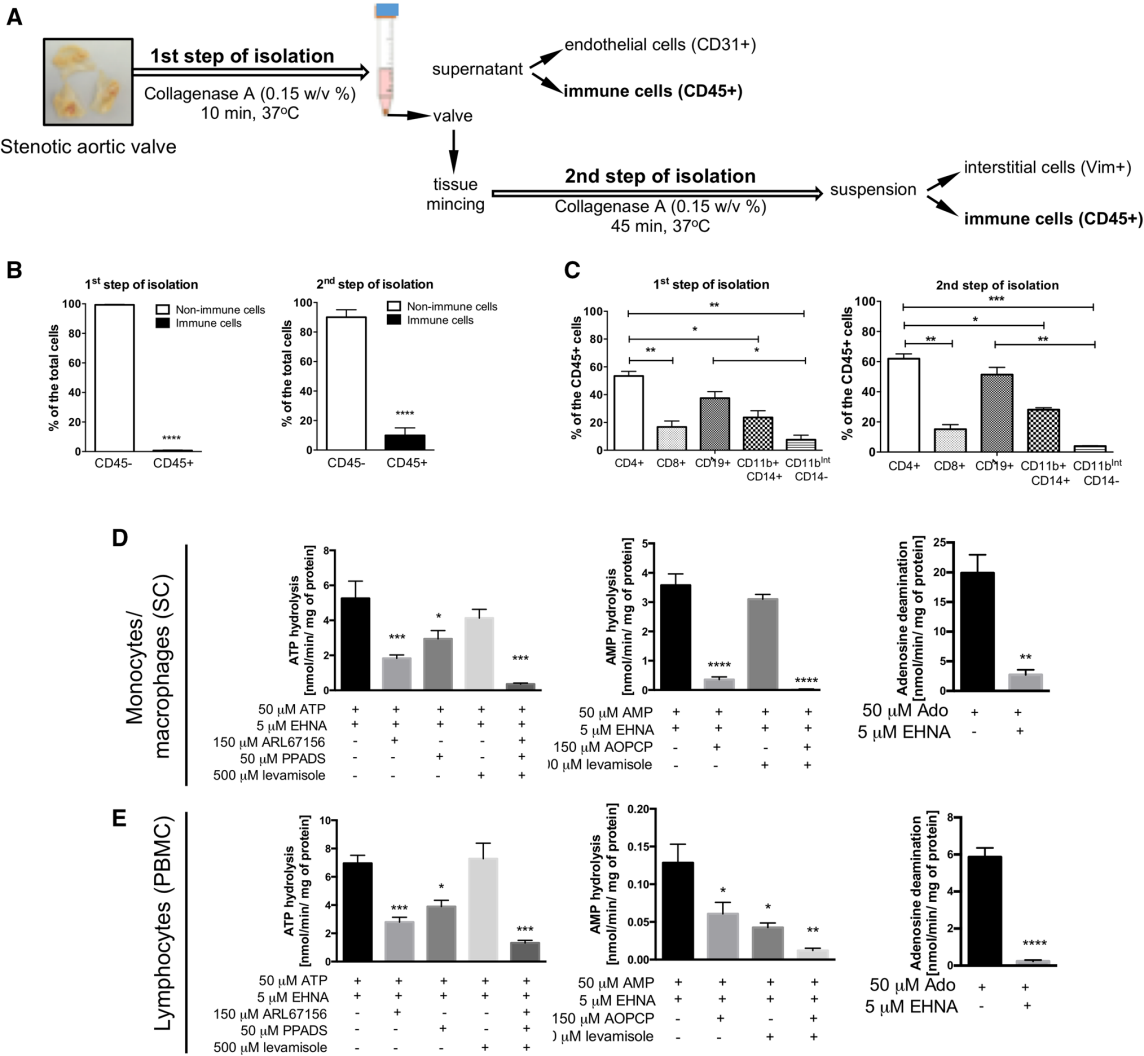
Stenotic aortic valve immune infiltrate is a smaller source of nucleotide-degrading ecto-nucleotidases but a larger of adenosine deaminase. Simplified protocol of stenotic aortic valve cell isolation, including endothelial cells (first step of isolation, vWF positive or CD31^high^ positive), interstitial cells (second step of isolation; Vimentin positive, Vim+) and immune cells (first and second step of isolation; CD45 positive, CD45+) (**a**). Flow cytometry analysis of CD45 positive cells (immune cells) as a percentage of total isolated cells after first step of isolation (cells located in the upper layers of the valve) and second step of isolation (cells located in the deeper layers of the valve) (**b**). The composition of stenotic aortic valve immune infiltrate (**c**) expressed as a percentage (%) of total CD45+ cells, including T helper cells (CD45+, CD4+), T cytotoxic cells (CD45+, CD8+), B cells (CD45+, CD19+), monocytes/macrophages (CD45+, CD11b+, CD14+) and granulocytes (CD45+, CD11b^int^, CD14−). The rates of ATP hydrolysis, AMP hydrolysis and adenosine deamination on the surface of human monocyte/macrophages (SC; **d**) and human peripheral blood mononuclear cells (PBMC = lymphocytes; **e**) and in the presence of ecto-enzyme inhibitors. Results are shown as mean ± SEM; *n *= 5–9, *p < 0.05, ***p < 0.001, ****p < 0.0001 vs. the first column by one-way Anova followed by Holm–Sidak post hoc test or Student *t* test

Then, we isolated stenotic aortic valve endothelial and interstitial cells (Fig. 5a, S4a) and immediately after isolation, we analyzed them with flow cytometry for CD39, CD73 and CD26 (ADA-binding protein). As first, we compared the levels of cell-surface proteins on stenotic aortic valve endothelial cells. The highest mean fluorescence intensity for CD31 (endothelial cell marker) positive cells expressed CD73, then CD39 and CD26 (Fig. S4b). In addition, the most of CD31 positive cells expressed on their surface CD73, about 40% expressed CD39 and only about 10% expressed CD26 (Fig. S4c). These results are in line with baseline levels of CD73 activity (Fig. 4b, c) and signal for this protein that colocalized with vWF in immunofluorescence (Fig. S4d–f).

Next, we expanded the analysis of hVIC. After the isolation of stenotic aortic valve interstitial cells (Fig. S4a), which were vimentin positive (Vim+), we gated them as myofibroblast-like cells, which were αSMA highly positive and bone sialoprotein (Sia) negative (Vim+, αSMA^high^, Sia−), transcient phenotype cells, which were Sia positive and αSMA intermediate positive (Vim+, αSMA^int^, Sia+) and osteoblast-like cells, which were Sia positive and αSMA negative (Vim+, αSMA−, Sia+) **(**Fig. S4g). Only activated myofibroblast-like VICs expressed significant amounts of CD39. While, myofibroblast-like VICs and transcient phenotype VICs barely had any CD39 on their surfaces. The mean fluorescence intensity for CD73 was higher on myofibroblast-like and transition state-VICs than in osteoblast-like VICs (Fig. S4h). These results could explained the changes in ecto-enzyme activities on VICs treated with osteogenic medium, where transition exclusively into osteoblast-like phenotype may lead to loss of CD39 and CD73 activity (Fig. 4e).

### The origin and activity of individual enzymes of extracellular nucleotide and adenosine metabolism in immune cells

In a stenotic aortic valve, besides the valvular cells, there is also an inflammatory infiltrate that could be a source of nucleotide- and adenosine-degrading ecto-enzymes. Immunofluorescence results revealed that fibrosa surface and calcification regions of stenotic aortic valves were a large source of CD45 and CD26 positive signal, which are a markers of immune cells (Fig. S5a, b). Immune cells (Fig. 5a) isolated from upper layers of stenotic valve during the first isolation step accounted for no more than 2% of all cells, while immune cells isolated from deeper layers during the second isolation step were around 10% of a total number of cells (Fig. 5b). In both cases, the dominant type of inflammatory cells were T helper cells (CD45+, CD4+), then B cells (CD45^+^, CD19^+^) and macrophages (CD45+, CD11b+, CD14−) (Fig. 5c). In contrast, isolates of inflammatory cells from all layers of the valve exhibited a small number of T cytotoxic cells (CD45+, CD8+) and granulocytes (CD45+, CD11b^int^, CD14−) (Fig. 5c).

Despite that immune cells are not a dominant cell type in stenotic aortic valves, they are still responsible for the origin of a certain pool of ecto-enzymes engaged in nucleotide and adenosine catabolism. As we have shown, the most significant in the number of cells and the presence of ecto-enzymes on their surfaces was the infiltrate of lymphocytes and monocytes/macrophages. Therefore, during functional assays, we estimated the rates of nucleotide and adenosine degradation on the surface of human peripheral blood mononuclear cells (PBMC), which are mostly lymphocytes [47] and on monocytes/macrophages (SC cell line). We also used ecto-enzyme inhibitors to identify activities of individual enzymes. On the surface of lymphocytes, we observed 60% of ATP hydrolysis inhibition after incubation with ARL67156, 30% of ATP hydrolysis inhibition after incubation with PPADS, and only 10% of ATP hydrolysis inhibition after using a levamisole (Fig. 5d), which means that ecto-nucleotidases (CD39 and eNPP1) as well as ALP are important in extracellular ATP removal in these cells. The effects of individual inhibitors on ATP hydrolysis was similar in monocytes/macrophages (Fig. 5d). In turn, AMP hydrolysis on lymphocytes was inhibited by 90% after incubation with AOPCP and about 10% after levamisole (Fig. 5e) that highlights the role of CD73 activity in AMP hydrolysis on lymphocytes. The rate of AMP hydrolysis on monocytes/macrophages was much lower than on lymphocytes and it was inhibited by about 50–60% after incubation with both AOPCP and levamisole (Fig. 5d). Adenosine deamination was inhibited by 80% on lymphocytes and by 90% on monocytes/macrophages after incubation with EHNA (Fig. 5d, e) and the activity of ADA1 (susceptible to inhibition by EHNA) on lymphocytes was the highest among all analyzed enzymes.

Further flow cytometry analysis of ecto-enzyme expression on immune cells isolated from stenotic aortic valves confirmed above results (Fig. S6). CD39 originated mainly from lymphocytes (B cells and T helper cells) and monocytes/macrophages. All immune cell subsets were a poor source of CD73, except a certain population of lymphocytes (B cells) and all analyzed immune cells revealed a high expression of an ADA-binding protein (CD26).

### Adenosine receptors in human aortic valves

**Fig. 6:**
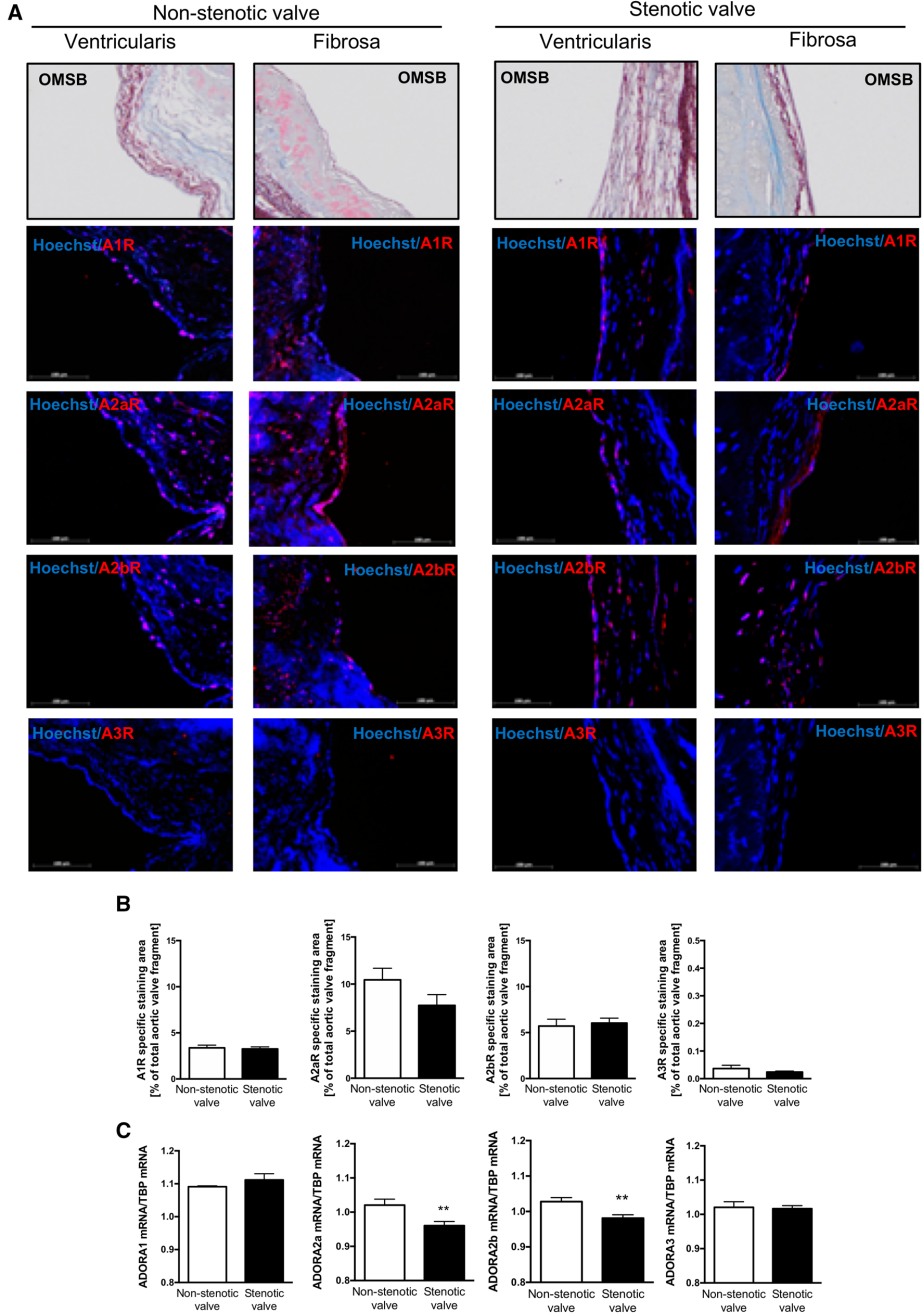
Adenosine receptors are widely express in human non-stenotic and stenotic aortic valves. Representative images of fibrosa and ventriculatis of non-stenotic and stenotic aortic valve (*n *= 3) stained with Orcein Mertius Scarlet Blue (OMSB) and representative images of matching sections stained by immunofluorescence (red signal) for four types of adenosine receptors (**a**). Scale bar = 100 μm. Quantitative analysis of A1R, A2aR, A2bR, A3R positive area that corresponds to the red signal (**b**). Fluorescence values of the negative control slices were substracted from the fluorescence value of the stained slices. Results are shown as mean ± SEM. Relative mRNA expression for four types of adenosine receptors in human non-stenotic (*n* = 6) and stenotic (*n* = 9) aortic valves (**c**). TBP mRNA was used for normalization. The average expression for each adenosine receptor normalized per TBP mRNA was estimated from measurements for three leaflets independently, in the sites free of calcification. Results are shown as mean ± SD; **p < 0.01 vs. non-stenotic valve by student *t* test

Since, ecto-enzymes that are engaged in nucleotide and adenosine metabolism play a key role in the bioavailability of adenosine in extracellular space for adenosine receptors, we determined which receptors are present in non-stenotic and stenotic aortic valves and which cells may be responsible for their origin. IF study (Fig. 6a, S7) revealed that the most abundant among adenosine receptors in both non-stenotic and stenotic aortic valves was receptor A2a (A2aR) (Fig. 6b). A2b and A1 receptors (A2bR, A1R) occurred in smaller amounts (Fig. 6b). While A3 receptor (A3R) was not observed (Fig. 6b). Comparing with OMSB histological staining (Fig. 6a) and vWF staining (Fig. S4d–f), all three adenosine receptors that were found in aortic valves colocalized with endothelial cells. Whereas, their presence within deeper layers of the valve depended on the type of valve. A2aR was observed throughout the cross-section of non-stenotic valve (Fig. 6a), while it was almost undetectable in the deeper layers of stenotic valves and in calcifications (Fig. S7a, b). In contrast, A2bR was also observed in the depths of the stenotic valve, including calcification areas (Fig. S7a, b). Since, IF approach is not well adapted to conclude the differences in protein levels, we measured mRNA expression for adenosine receptors. This analysis revealed that expression of both, A2aR and A2bR was diminished in not calcified fragments of stenotic valves compared to non-stenotic (Fig. 6c).

### Pharmacological modulation of adenosine metabolism is beneficial in mouse model of CAVD via adenosine receptor dependent mechanisms

**Fig. 7:**
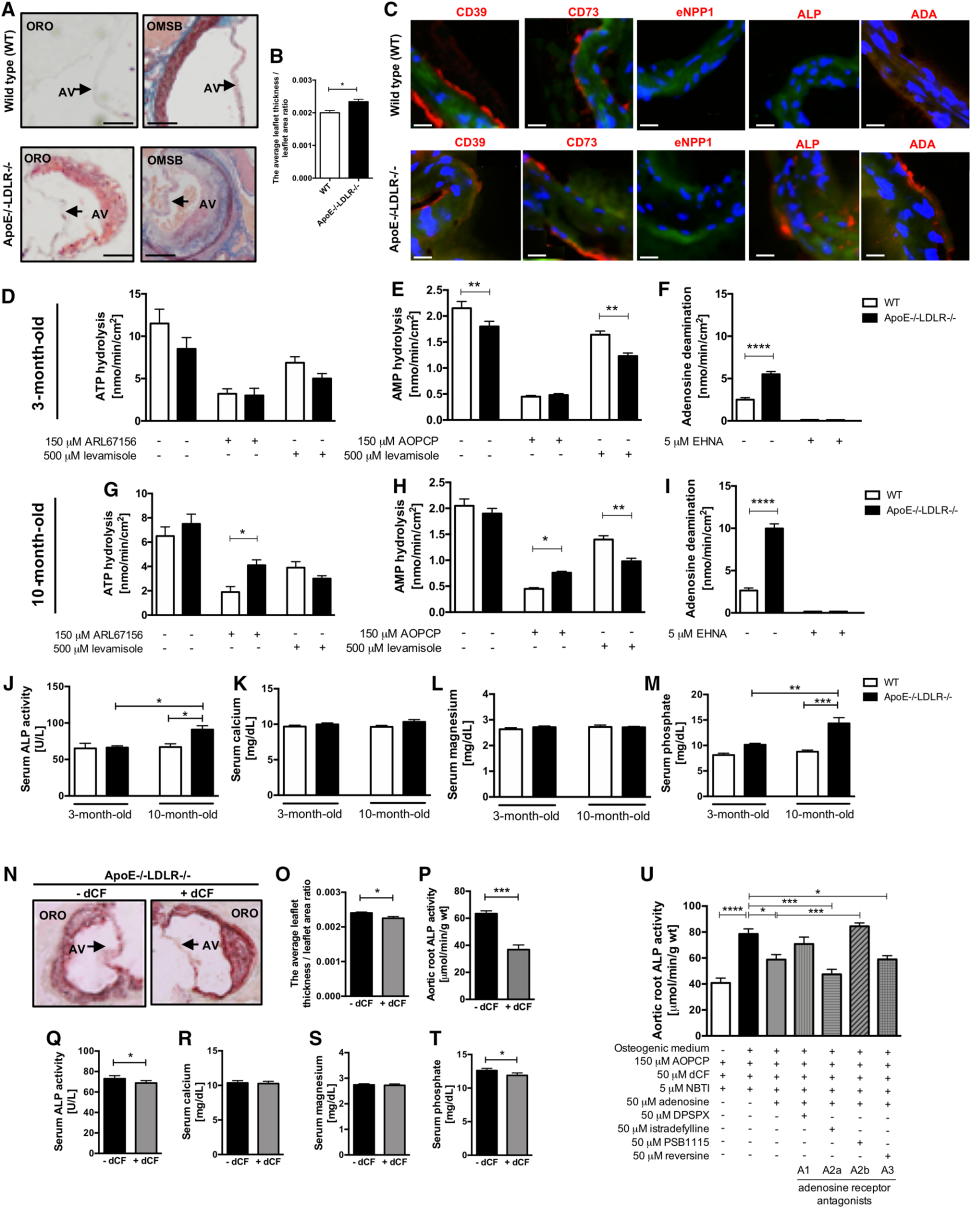
Modulation of adenosine metabolism by deoxycoformycin substantially decreases aortic valve thickness and reduces markers of calcification in a mouse model of CAVD. Representative images of 6-month old male wild type (WT) and ApoE−/−LDLR−/− mice aortic valve stained with Oil Red O (ORO) and Orcein Mertius Scarlet Blue (OMSB), *AV* aortic valve (**a**). The ratio of average leaflet thickness measured in 3 different places of aortic valve leaflet to leaflet area of WT and ApoE−/−LDLR−/− mice stained with ORO (**b**). Representative images of WT and ApoE−/−LDLR−/− mice aortic valve stained with immunofluorescence (red signal) for CD39, CD73, eNPP1, ALP, ADA (**c**). Results are shown as mean ± SEM; *n *= 3; *p < 0.05 vs. non-stenotic valve by Mann–Whitney test. The rates of ATP hydrolysis (**d**, **g**), AMP hydrolysis (**e**, **h**) and adenosine deamination (**f**, **i**) on the surface of male 3-month-old (**d**–**f**) and 10-month-old (**g**–**i**) WT and ApoE−/−LDLR−/− mice aortic roots and in the presence of ecto-enzyme inhibitors. Serum ALP activity (**j**), calcium (**k**), magnesium (**l**) and phosphate concentration (**m**). Results are shown as mean ± SEM; *n *= 4–6; *p < 0.05, **p < 0.01, ***p < 0.001, ****p < 0.0001 vs. WT by one-way Anova followed by Holm-Sidak post hoc test. Representative images of aortic valve of 6-month old male ApoE−/−LDLR−/− mice treated intraperitoneally with saline or 0.2 mg/kg deoxycoformycin (dCF) twice weekly for 2 months stained with Oil Red O (ORO), *AV* aortic valve (**n**). The ratio of average leaflet thickness measured in three different places of aortic valve leaflet to leaflet area of ApoE−/−LDLR−/− mice treated with saline or dCF stained with ORO (**o**). Aortic root ALP activity (**p**), serum ALP activity (**q**), calcium (**r**), magnesium (**s**) and phosphate (**t**) concentration. Results are shown as mean ± SEM; *n *= 5, *p < 0.05, ***p < 0.001 vs. saline-treated ApoE−/−LDLR−/− mice (−dCF) by Student’s *t* test. Wild type mice aortic root ALP activity after 96 h treatment with osteogenic medium with adenosine (50 μm) and adenosine receptor antagonists (50 μm) in the presence of 150 μm AOPCP, 5 μm dCF and 5 μm NBTI. Results are shown as mean ± SEM; *n *= 5, *p < 0.05, ***p < 0.001, ****p < 0.0001 by one-way Anova followed by Holm–Sidak post hoc test

In current study, patients with CAVD demonstrated abnormalities in serum lipid profile that correlated with the activities of adenosine-metabolizing enzymes, especially with eADA activity (Fig. 2). To analyze the effect of adenosine metabolism pharmacological modulation, C57Bl/6 J mouse double knock-out for ApoE and LDLR genes (ApoE−/−LDLR−/−) were characterized and used. In histological analysis, aortic valves of ApoE−/−LDLR−/− mice were thicker than valves obtained from wild types (WT) (Fig. 7a) and showed the increased ratio of leaflet thickness to leaflet area (Fig. 7b). In immunofluorescence, ApoE−/−LDLR−/− mice aortic valves expressed substantial amounts of ALP and ADA (Fig. 7c). It was consistent with aortic root ecto-enzyme activities (Fig. 7d–i). We observed higher rate of AMP hydrolysis in old ApoE−/−LDLR−/− mice in the presence of AOPCP and hence increased ALP activity, which was not shown in young mice (Fig. 7e, h). The activity of aortic root adenosine deamination was heightened in both age groups of ApoE−/−LDLR−/− mice in comparison to WT, but this increase was more pronounced in older mice (Fig. 7f, i). We also demonstrated higher serum ALP activity and phosphate concentration in 10-month-old ApoE−/−LDLR−/− mice, with no changes in serum calcium and magnesium concentration (Fig. 7j–m). Using previously published protocol of 2-month treatment with specific adenosine deaminase inhibitor, deoxycoformycin (dCF) [29], we observed a decrease in the thickness of ApoE−/−LDLR−/− mice aortic valve (Fig. 7n, o), reduced aortic root and serum ALP activity (Fig. 7p, q) and serum phosphate concentration (Fig. 7t). Using WT mice aortic roots treated for 96 h with osteogenic medium, we examined the effects of adenosine and adenosine receptor antagonists on the development of calcification (Fig. 7u). Although exogenous adenosine in the presence of AOPCP, dCF and NBTI decreased ALP activity in the presence of osteogenic medium, also A2a receptor antagonist, counteracted the increase in ALP activity stimulated by high phosphate medium. In turn, the increased calcification rate in the presence of adenosine was found by blocking A1 and particularly A2b receptors, which implied their anti-stenotic effect.

## Discussion

This study demonstrates an abnormal extracellular nucleotide metabolism in CAVD, which comprises a number of changes in ecto-enzyme activities on a variety of cell types. Consequently, stenotic aortic valves originated from CAVD patients were characterized by reduced levels of extracellular ATP removal and impaired production of adenosine. Moreover, already reduced levels of extracellular adenosine were immediately degraded further due to an elevated rate of adenosine deamination.

For the first time, we thoroughly analyzed the entire aortic valve surfaces and revealed that the above metabolic pattern was observed only on the fibrosa surface of stenotic aortic valve. Nucleotide and adenosine conversion rates measured in this particular location of the valve significantly correlated with the severity and risk factors of CAVD, such as hyperlipidemia, hypertension, and thrombosis.

Valvular activities of nucleotide-degrading ecto-nucleotidases negatively correlated with aortic jet velocity, mean transvalvular gradient and mineral deposit concentration in aortic valves. In turn, adenosine deamination tended to positively correlate with valvular phosphate concentration. Fibrosa surface AMP hydrolysis also negatively correlated with serum LDL cholesterol, while strong positive correlations were observed between adenosine deamination and serum lipid parameters. It follows that hyperlipidemia accompanies the removal of adenosine from human aortic valve surface. The similar metabolic pattern consisted of decreased extracellular AMP hydrolysis and increased adenosine deamination was observed in aortic roots of a mouse model of CAVD with altered lipid metabolism (ApoE−/−LDLR−/− mice). To underline the causality between aortic valve disease and differential expression of adenosine-metabolizing ecto-enzymes, we performed an interventional experiment using adenosine deaminase inhibitor in our mouse model of CAVD. Deoxycoformycin substantially decreased aortic valve thickness in ApoE−/−LDLR−/− mice and reduced markers of calcification.

In this work, the increased adenosine deaminase activity measured on the fibrosa surface of human aortic valve also significantly correlated with systolic blood pressure in CAVD patients. Hypertension could be an effect of increased vascular adenosine degradation as adenosine plays an important role in vascular dilatation. [48, 49] Due to turbulent blood flow on the aortic side of the valve and blood retention during valve closure, this specific part of the valve is prone to immune cell attachment and infiltration followed by the endothelial injury and calcification [50, 51] In our previous studies, we determined ecto-adenosine deaminase activity as a marker of endothelial activation and immune cell infiltration and these conditions could be a direct cause of eADA increase on the fibrosa surface of stenotic aortic valve. Also in the current study, we revealed that lymphocytes and monocytes/macrophages isolated from stenotic aortic valves were characterized by a high expression of ADA-binding protein. Whereas, functional assays using these cells revealed high eADA activity that via reduction of protective adenosine, favors pro-inflammatory milieu. On the other hand, increased eADA activity could also be a reflection of endothelial activation, since such pathological conditions as atherogenic lipoproteins enhance its endothelial activity that may explain highly positive correlations of fibrosa surface eADA activity with serum lipid profile parameters. [29, 52, 53].

Substrates for extracellular enzymes are released by different cells in the entire circulation, including stimulated cells localized within the aortic valve. [11] In addition, the availability of particular nucleotide catabolism ecto-nucleotidases is variable and depends on cell type and each cell’s specific functions. In the cardiovascular system, the most important role in the extracellular ATP catabolism is attributed to the family of ecto-nucleoside triphosphate diphosphohydrolases (eNTPDases). As it has been shown so far, the major member of this family, eNTPD1/CD39 is predominantly expressed in the vasculature by endothelial cells and vascular smooth muscle cells (VSMC). [54] Another enzyme involved in extracellular ATP degradation is ecto-nucleotide pyrophosphatase/phosphodiesterase 1 (eNPP1) [21] that has been found at high levels in valvular interstitial cells during CAVD. [22] In our study, we confirmed the presence of eNPP1 in aortic valves by immunofluorescence and found its activity on the surface of hVIC and to some extent on inflammatory cells that infiltrate stenotic aortic valve. However, these cells and, above all, valvular endothelial cells expressed also CD39 in our immunofluorescence, flow cytometry, and biochemical studies. Considering previously described increase in the eNPP1 expression in CAVD [22], we assume that the decreased ATP hydrolysis on the fibrosa surface of stenotic valve observed in our study is the effect of diminished CD39 activity, which expression was reduced in stenotic valves. In recent studies, we have shown the decreased activity and protein level of CD39 in the homogenates of stenotic aortic valves using functional assays, immunohistochemistry [46] and proteomics [55]. In addition, we demonstrated a lower level of CD39 on hVIC ongoing differentiation into osteoblast-like. Based on controlling extracellular purinergic gradient, the reduction in CD39 activity can have a number of consequences in CAVD development, particularly associated with thrombosis and inflammation. In our study, ATP removal from fibrosa surface of aortic valve positively correlated with anti-thrombotic phenotype in CAVD patients. Whereas, other studies have shown that systemic administration of CD39 minimized injury-induced platelet deposition and leukocyte recruitment, [56, 57] and CD39 knockout mice decreased neointimal formation associated with impaired VSMC migration. [19] Moreover, the decreased CD39 activity can promote extracellular ATP accumulation within the aortic valve, but a role of this nucleotide in CAVD is fairly controversial. On the one hand, ATP via specific subtypes of P2 purinergic receptor triggered inflammation, endothelial activation and hVIC calcification. [9, 58] While other study presented that the depletion of extracellular ATP due to high level of eNPP1 led to a shutdown of the PI3 K/Akt pathway, which ensured in normal condition the survival of VICs, preventing VIC apoptosis-mediated mineralization. [22] Moreover, extracellular ATP via eNPP1 activity could be a source of inorganic pyrophosphate (PPi), a potent inhibitor of passive calcification. [59] However, PPi can be further degraded to inorganic phosphate (Pi) that is an inductor of calcification via tissue non-specific alkaline phosphatase (TNAP). [45, 60] Taken together, these differential effects evoked by extracellular ATP cannot be considered separately and should be analyzed in proper models of CAVD were dynamic changes in endothelial condition, inflammation and mineralization occur.

Another important enzyme of nucleotide metabolism that supports anti-inflammatory and anti-thrombotic milieu is adenosine-producing ecto-5′nucleotidase (CD73). [61, 62] CD73 has been found in a variety of tissues, including abundant activity in vascular endothelium. [63] We have demonstrated that CD73 was the most important ecto-enzyme responsible for AMP hydrolysis on the surface of valvular endothelial and interstitial cells, as well as on the immune cells isolated from stenotic aortic valves. However, the total rate of AMP to adenosine hydrolysis and hence CD73 activity was much higher on valve cells than on immune cells isolated from stenotic valves. Our immunohistochemical [46] and current immunofluorescence data revealed that a part of the signal for CD73 can accumulate around the areas of calcification, while in not calcified sections of stenotic aortic valves, we observed a rather weak signal for this protein and its reduced activity in comparison to non-stenotic valves. Higher expression of CD73 around the calcification areas may result from VICs at transition state from myofibroblast-like to osteoblast-like cells, which expressed the highest levels of CD73 in our flow cytometry analysis among all VICs isolated from stenotic valves and could be the effect of the active process where inflammatory cells secrete mediators to activate and differentiate VICs. Whereas, lower activity of CD73 in not calcified sections of fibrosa surface of stenotic valves may be due to decreased expression of this enzyme on valvular endothelial cells or valve fibrosis and reduced cellular components within deeper layers of the aortic valve. In contrast to our results, it has been reported previously that stenotic aortic valves revealed overexpression of CD73. [10] However, the analysis could be overestimated due to the accumulation of CD73 protein around calcifications.

CD73-derived adenosine performs many critical functions in vasculature calcification, including the inhibition of TNAP, and thus PPi to Pi conversion. [64] In this context, increased activities of both eNPP1 and TNAP in stenotic valves provide a sequential breakdown of ATP to the formation of Pi, while decreased CD73 activity impairs the formation of adenosine from remaining AMP, which further stimulates calcification. Patients with mutations in CD73 gene exhibited ectopic calcification within the cardiovascular system that was dependent on the lack of TNAP-inhibiting adenosine. [65] These reports suggest that inhibitors of TNAP, such as adenosine, could be considered as potential preventive strategies in CAVD and supporting this hypothesis, we observed decreased serum and aortic root ALP activity in ApoE−/−LDLR−/− mice treated with adenosine deaminase inhibitor.

However, the effects of adenosine on vasculature calcification could be also dependent on activated adenosine receptors. It has been shown previously that the stimulation of A1 receptors (A1R) promoted an anti-mineralizing response, whereas A2a receptor (A2aR) activation had the opposite effect on human VIC calcification. [10] Adenosine was also attributed to have pro-stenotic properties by the stimulation of ovine VIC degeneration by A2aR and A2b receptor (A2bR) activation. [66] On the contrary, treatment with A2bR agonist significantly reduced the calcification volume of progenitor cell-derived teratomas from patients with CD73 deficiency. [65] Also in our recent work, we have shown that CD73−/− mice spontaneously developed aortic valve dysfunction increasing peak aortic flow and valvular deposit concentration. [67] In the current study, we treated mice aortic roots with the osteogenic medium in the presence of adenosine and adenosine receptor antagonists and observed that beneficial effects of adenosine were dependent on both A1R and A2bR activation, while the stimulation of A2aR promoted mineralization. It is crucial to emphasise that the stimulation of A2bR can cause many positive outcomes, including endothelial protection [68], lipid-lowering [69] and anti-inflammatory [70] effects, which can mediate its anti-stenotic properties. It should be also noted that A2bR is activated only by high adenosine concentration (micromolar) and, therefore, A2bR-dependent effects will be triggered when the production of adenosine is maintained by ecto-nucleotidases and it is not excessively degraded by eADA. [71] It is also essential that complex aortic valve microenvironment is composed of many cell types including endothelial, interstitial, immune and incoming progenitor cells, which can influence each other to the development of inflammation and calcification by purinergic-dependent and independent mechanisms. [72].

## Conclusion

Complex purinergic signaling pathways in CAVD involve the deregulation of many ecto-enzyme activities and adenosine receptor expression, which originate from various types of cells that build and pathologically infiltrate aortic valves. Hence, the wide-spectrum approach should be used both for the analysis of purinergic signaling in CAVD and for the study of potential therapeutic effects of drugs regulating the extracellular pathways of nucleotide and adenosine metabolism. It is clear that enzymes engaged in the extracellular nucleotide cascade play a significant role in all stages of CAVD, from controlling endothelial damage, through leukocyte infiltration, accumulation of foam cells and secretion of pro-inflammatory mediators to osteoblastic differentiation. Thus, adequate activities of nucleotide and adenosine-regulating ecto-enzymes can be viewed as specific “switches” that shift ATP-driven valvular dysfunction and degeneration toward the states mediated by adenosine, which in turn are dependent on activated adenosine receptors.

### Study limitations

Some limitations of our study should be acknowledged. First, the sample size was rather small, thus demanding cautious data interpretation.

Second, although, as discussed above, various findings suggest an important relationship between stenotic valve nucleotide metabolism with serum lipid profile, coagulation parameters, and blood pressure, CAVD patients are under pharmacological intervention that affects these parameters. In our earlier work, we demonstrated that e.g. statins can lower the activity of eADA in endothelial cells. [29, 73] Therefore, demonstration of the impact of individual drugs on extracellular nucleotide metabolism enzyme activities in VEC, VIC and immune cells is crucial and we are currently investigating this aspect.

Third, employing mouse models are not immediately transferable to humans because of existing differences in humans and mice, including changes in lipid metabolism and serum cholesterol levels.

Fourth, discrepancies in nucleotide and adenosine metabolism between species should be considered. For example, ADA2-dependent mechanisms of immune cell differentiation cannot be examined in mice due to the lack of ADA2 in rodents.

## Electronic supplementary material

Below is the link to the electronic supplementary material.

Supplementary material 1 (DOCX 7613 kb)

## Abbreviations

ATP: Adenosine triphosphate
ADP: Adenosine diphosphate
AMP: Adenosine monophosphate
PPi: Inorganic pyrophosphate
Pi: Inorganic phosphate
CD39: Ecto-nucleoside triphosphate diphosphohydrolase 1
eNPP1: Ecto-nucleotide pyrophosphatase/phosphodiesterase 1
CD73: Ecto-5′-nucleotidase
TNAP: Tissue nonspecific alkaline phosphatase
eADA: Ecto-adenosine deaminase
dCF: 2′Deoxycoformycin (adenosine deaminase inhibitor)
VEC/ hVEC: Valvular endothelial cells
VIC/ hVIC: Valvular interstitial cells
aVIC: Activated VIC
obVIC: Osteoblast-like VIC
a/obVIC: Transient phenotype VIC
LDL: Low density lipoproteins
oxLDL: Oxidized LDL
α-SMA: Smooth muscle cells alpha actin
A1R: Adenosine A1 receptor
A2aR: Adenosine A2a receptor
A2bR: Adenosine A2b receptor
A3R: Adenosine A3 receptor
AOPCP: 5′-(α,β-methylene)diphosphate
AP: Alkaline phosphatase
ARL67156: 6-*N*,*N*-Diethyl-β-γ-dibromomethylene-d-adenosine-5′-triphosphate trisodium salt hydrate
AVR: Aortic valve replacement
CAVD: Calcific aortic valve disease
EHNA: Erythro-9-(2-hydroxy-3-nonyl)adenine
FBS: Fetal bovine serum
HBSS: Hanks balanced salt solution
HE: Hematoxylin and eosin staining
HPLC: High performance liquid chromatography
NBTI: *S*-(4-Nitrobenzyl)-6-thioinosine
OMSB: Orcein martius scarlet blue staining
PBMC: Peripheral blood mononuclear cells
PBS: Phosphate buffered saline
PPADS: Pyridoxal phosphate-6-azo(benzene-2,4-disulfonic acid) tetrasodium salt hydrate
qVIC: Quiescent VIC
TAVI: Transcatheter aortic valve implantation
TR: Masson’s trichrome staining
VSMC: Vascular smooth muscle cells
vWF: von Wilebrant factor
