# Supplementary material for: Nucleotide ecto-enzyme metabolic pattern and spatial distribution in calcific aortic valve disease; its relation to pathological changes and clinical presentation
Source: Clin Res Cardiol. 2019 May 29;109(2):137–60. doi: 10.1007/s00392-019-01495-x (PMC6989624; doi:10.1007/s00392-019-01495-x)
Supplement: Supplementary file 1 — Supplementary material 1 (DOCX 7613 kb) [file 392_2019_1495_MOESM1_ESM.docx]

**Nucleotide ecto-enzyme metabolic pattern and spatial distribution in calcific aortic valve disease; its relation to pathological changes and clinical presentation**

**Supplementary material**

Barbara Kutryb-Zajac^1^, Patrycja Jablonska^1^, Marcin Serocki^2^, Alicja Bulinska^1^, Paulina Mierzejewska^1^, Daniela Friebe^3^, Christina Alter^3^, Agnieszka Jasztal^4^, Romuald Lango^5^, Jan Rogowski^6^, Rafal Bartoszewski^2^, Ewa M. Slominska^1^, Stefan Chlopicki^4^, Jürgen Schrader^3^**,** Magdi H. Yacoub^7^, Ryszard T. Smolenski^1^

**Supplemental material and methods**

1. **Determination of valve deposits compounds concentrations**

Calcium content was estimated by Arsenazo III method, which relies on the formation of blue-purple complex at neutral pH. [1] Valvular magnesium concentration was analyzed using Calmagite, which forms a red complex with Mg^2+^ in an alkaline solution. [2] In turn, phosphate content was determined through a production of the green complex with malachite green molybdate under acidic conditions. [3] The intensity of solution decoloration was measured spectrophotometrically (Microplate Spectrophotometer Synergy HT, BioTek Instruments, Inc., Winoosk, VT) at 630 nm for Ca^2+^ and PO_4_^3-^ and 490 nm for Mg^2+^. Results were expressed as mg of calcium, magnesium or phosphate per wet weight of tissue (mg/g wt).

1. **Histological analysis**

Images were acquired using a Dot Slide automatic scanning station (Olympus, Japan), stored as tiff files and analyzed automatically by the Image Browser software (Carl Zeiss). Areas of calcification were assessed in 6 cross-sections per each valve stained with TR and OMSB. Data were shown as the mean area of calcification expressed as the percentage of total aortic valve area.

1. **Imunofluorescence analysis**

Human CD39 and CD73 were stained using mouse anti-human CD39 (*Novus*) and mouse anti-human CD73 (*Novus*) primary antibody, followed by a Cy3-conjugated goat anti-mouse secondary antibody (*JacksonImmuno*). Human eNPP1, ALP, ADA, vWF, CD26, CD45, A1R, A2aR, A2bR and A3R were stained using a rabbit anti-human eNPP1 (*Novus*), rabbit anti-human ALP (*Novus*), rabbit anti-human ADA (*Proteintech*), rabbit anti-human vWF (*Proteintech*), rabbit anti-human CD26 (*Genetex*), rabbit anti-human CD45 (*Genetex*), rabbit anti-human A1R (*Novus*), rabbit anti-human A2aR (*Novus*), rabbit anti-human A2bR (*Novus*), rabbit anti-human A3R primary antibody (*Novus*), followed by a Cy3-conjugated goat anti-rabbit secondary antibody (*JacksonImmuno*). Human imentin was stained using mouse anti-human vimentin conjugated with AlexaFluor488 (*Novus*). Mouse CD39, CD73, eNPP1, ADA and ALP were stained using rat anti-mouse CD39 (*Abcam*) and rat anti-mouse CD73 (*Abcam*) primary antibody, followed by a Cy3-conjugated goat anti-rat secondary antibody (*JacksonImmuno*), rabbit anti-mouse eNPP1 (*Novus*), rabbit anti-mouse ALP (*Novus*), rabbit anti-mouse ADA (*Proteintech*) primary antibody, followed by a Cy3-conjugated goat anti-rabbit secondary antibody (*JacksonImmuno*).

1. **Gene expression**

TaqMan probes ids used were: CD73 - Hs00159686_m1; CD39 - Hs00969559_m1; ADA - Hs01110945_m1; ADORA1 Hs00181231_m1; ADORA2a - Hs00169123_m1; ADORA2b - : Hs00386497_m1; ADORA3 - Hs00252933_m1.

1. **Peripheral blood mononuclear cells isolation**

To isolate peripheral blood mononuclear cells (PBMC) from healthy human donors, a layer of 3 mL Histopaque 1.077 g/mL (*Sigma, USA*) was applied to the layer of 3 mL Histopaque 1.119 g/mL (*Sigma, USA*) in a 15-mL conical centrifuge tube. Blood was applied to the Histopaque 1.077 g/mL layer and it was centrifuged at 400 x *g* for 30 min at room temperature. After centrifugation, the upper layer was aspirated to within 0.5 cm of the opaque interface containing mononuclear cells and discarded. The opaque interface was transferred into a clean conical centrifuge tube and washed with 0.9 % NaCl containing 5 mM EDTA. After centrifugation (250 x *g*, 10 min), the supernatant has been discarded and the cell pellet was resuspended with HBSS.

1. **Determination of alkaline phosphatase activity on the surface of mice aortic roots**

ALP activity on the surface of mice aortic roots was measured in HBSS with 50 μM AMP as a substrate, 150 μM AOPCP as CD73 inhibitor and 5 μM EHNA as ADA1 inhibitor to prevent further degradation of adenosine to inosine. After 30 min incubation at 37°C, AMP and adenosine concentrations were assessed using HPLC as described earlier [4] and ALP activity has been expressed as μmol/min/g of wet tissue.

1. **Flow cytometry analysis**

Cells for flow cytometry analysis were stained with following antibodies: anti-CD31-PE-Cy7, WM59 (*eBioscience*), anti-vimentin-AF488, RV203 (*Novus*), anti-bone sialoprotein-PE-Cy7 polyclonal (*Biorbyt*), α-SMA-eFluor660, 1A4 (eBioscience), anti-CD45-APC, 30-F11 (*BD Bioscience*), anti-CD4-PerCP-Cy5.5, RM405 (*eBioscience*), anti-CD8a-APC-H7, 53-6.7 (*BD Bioscience*), anti-CD19-PE-TR, SJ25-C1 (*LifeSpan BioSciences*), anti-CD11b-PE M1/70 (*BD Bioscience*), anti-CD14-AF488, M5E2 (*StemCell*), anti-CD73-FITC, 496406 (*R&D Systems*), anti-CD39-PE-Cy7, 24DMS1 (*eBioscience*), anti-CD26-PE 2A6 (*eBioscience*). Cell measurements were performed with a FACSCAnto II flow cytometer (BD Bioscience). For analysis, the placement of gates was based on fluorescence minus one (FMO) controls. The minimum number of events used to define a cell population was 150. The analysis was performed on individual aortic valves.

**References**

1. Michaylova V, Ilkova P (1971) Photometric determination of micro amounts of calcium with arsenazo III. Anal Chim Acta 53:194–198

2. Chauhan UPS, Ray Sarkar BC (1969) Use of calmagite for the determination of traces of magnesium in biological materials. Anal Biochem 32:70–80

3. Feng J, Chen Y, Pu J, et al (2011) An improved malachite green assay of phosphate: Mechanism and application. Anal Biochem 409:144–149

4. Smolenski RT, Lachno DR, Ledingham SJM, Yacoub MH (1990) Determination of sixteen nucleotides, nucleosides and bases using high-performance liquid chromatography and its application to the study of purine metabolism in hearts for transplantation. J Chromatogr B Biomed Sci Appl 527:414–420. https://doi.org/10.1016/S0378-4347(00)82125-8

**Supplemental figures**

**
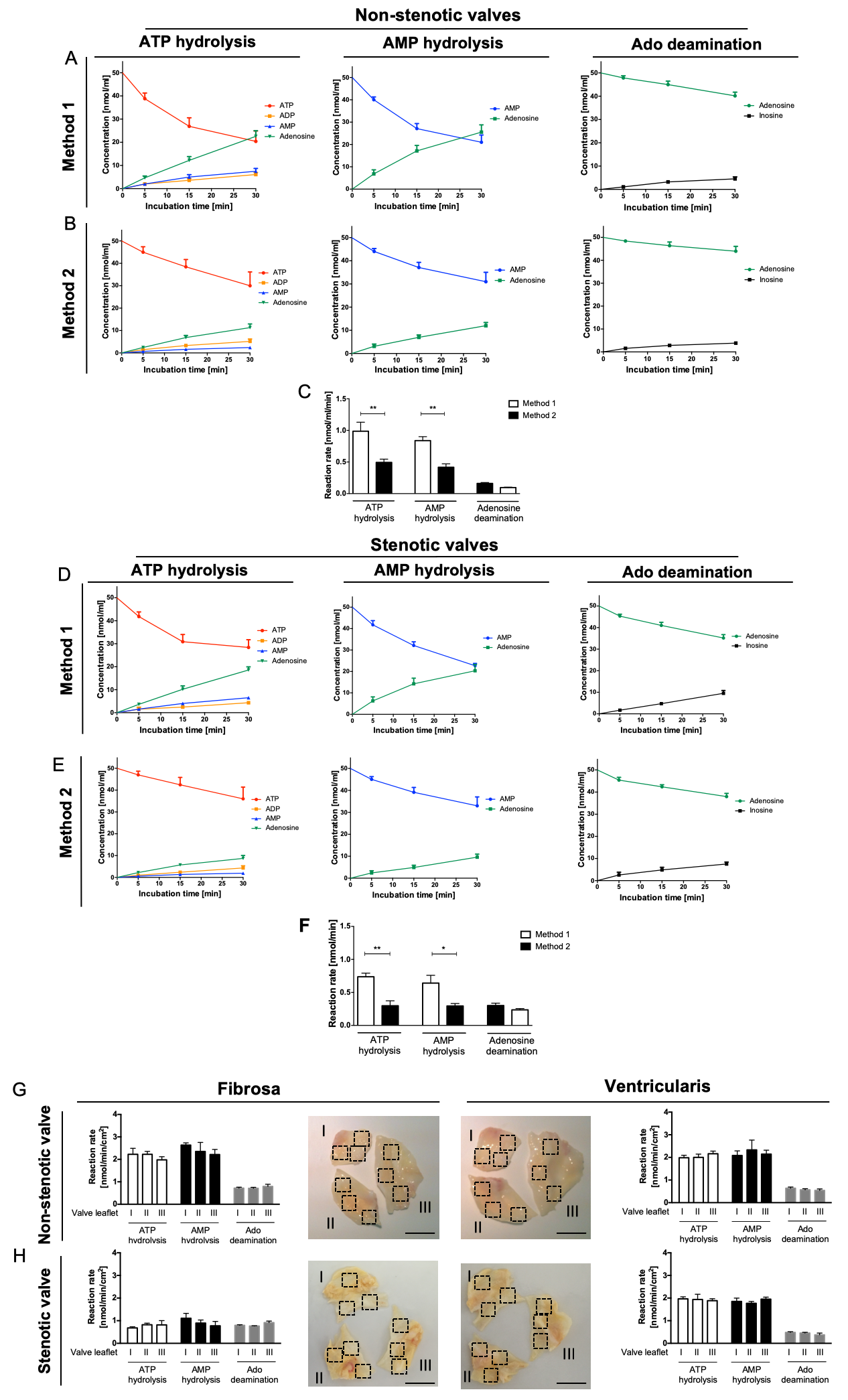
**

**Figure S1. Activities of nucleotide metabolism ecto-enzymes are maintained on both sides of non-stenotic and stenotic aortic valves and do not differ between leaflets within the same aortic valve.** The concentration of substrates and products during a test **(A, B, D, E)** and calculated rates of ATP hydrolysis, AMP hydrolysis and adenosine deamination **(C, F)** after 30 min incubation of the entire fragment (**Method 1**) of the non-stenotic aortic valve (**A**) or stenotic aortic valve **(D)** and after the exposure of the fibrosa surface (**Method 2**) of non-stenotic aortic valve (**B**) or stenotic aortic valve **(E)** to Hanks Balanced Salt Solution with 50 µM ATP, AMP or adenosine. Results are shown as mean ± SEM, *n*=4; **p*<0.01, ***p*<0.01 vs. Method 1 by two-way Anova followed by Sidak *post-hoc* test. Rates of ATP hydrolysis, AMP hydrolysis and adenosine deamination on the fibrosa and ventricularis surfaces of non-stenotic **(G)** and stenotic **(H)** aortic valve leaflets. Results are shown as mean activity for each leaflet determined on the surface of three regions of the leaflet (non-calcified regions have been chosen for stenotic valve) ± SEM, *n*=9 (independent analysis of 3 aortic valves) by one-way Anova followed by Holm-Sidak *post-hoc* test.


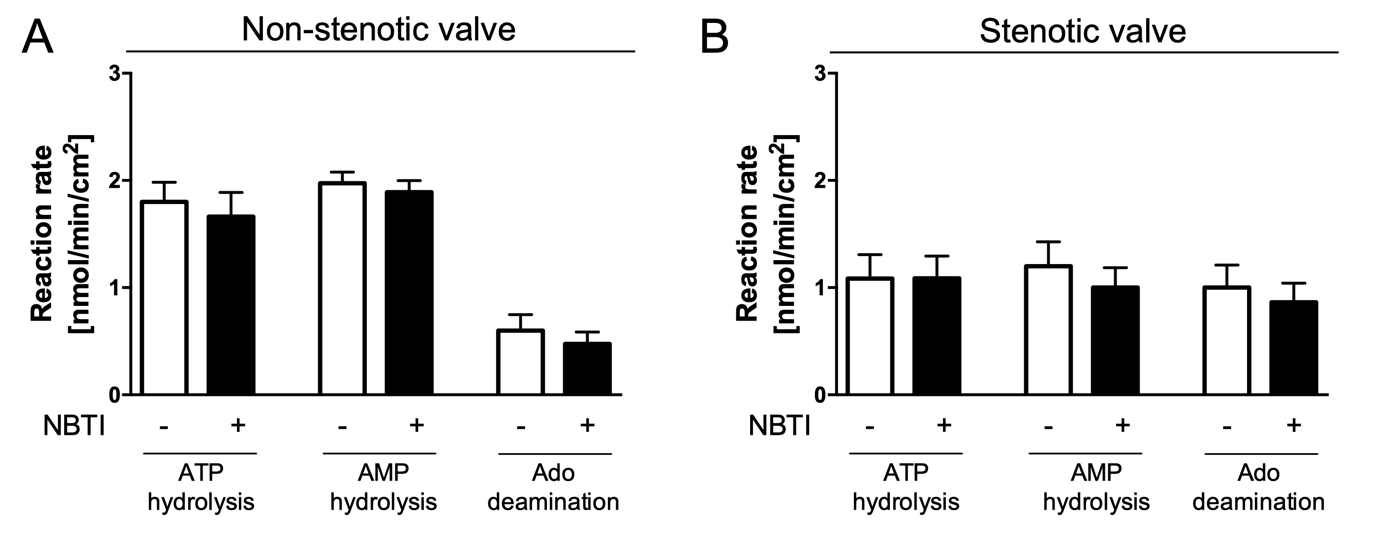


**Figure S2. Extracellular nucleotide metabolism in the aortic valve is mediated by cell-surface ecto-enzymes.** Rates of ATP hydrolysis, AMP hydrolysis and adenosine deamination on the fibrosa surface of non-stenotic **(A)** and stenotic aortic valve **(B)** after incubation with a nucleoside transport inhibitor, NBTI (S-4-Nitrobenzyl)-6-thioinosine). Results are shown as mean ± SEM, *n*=4.


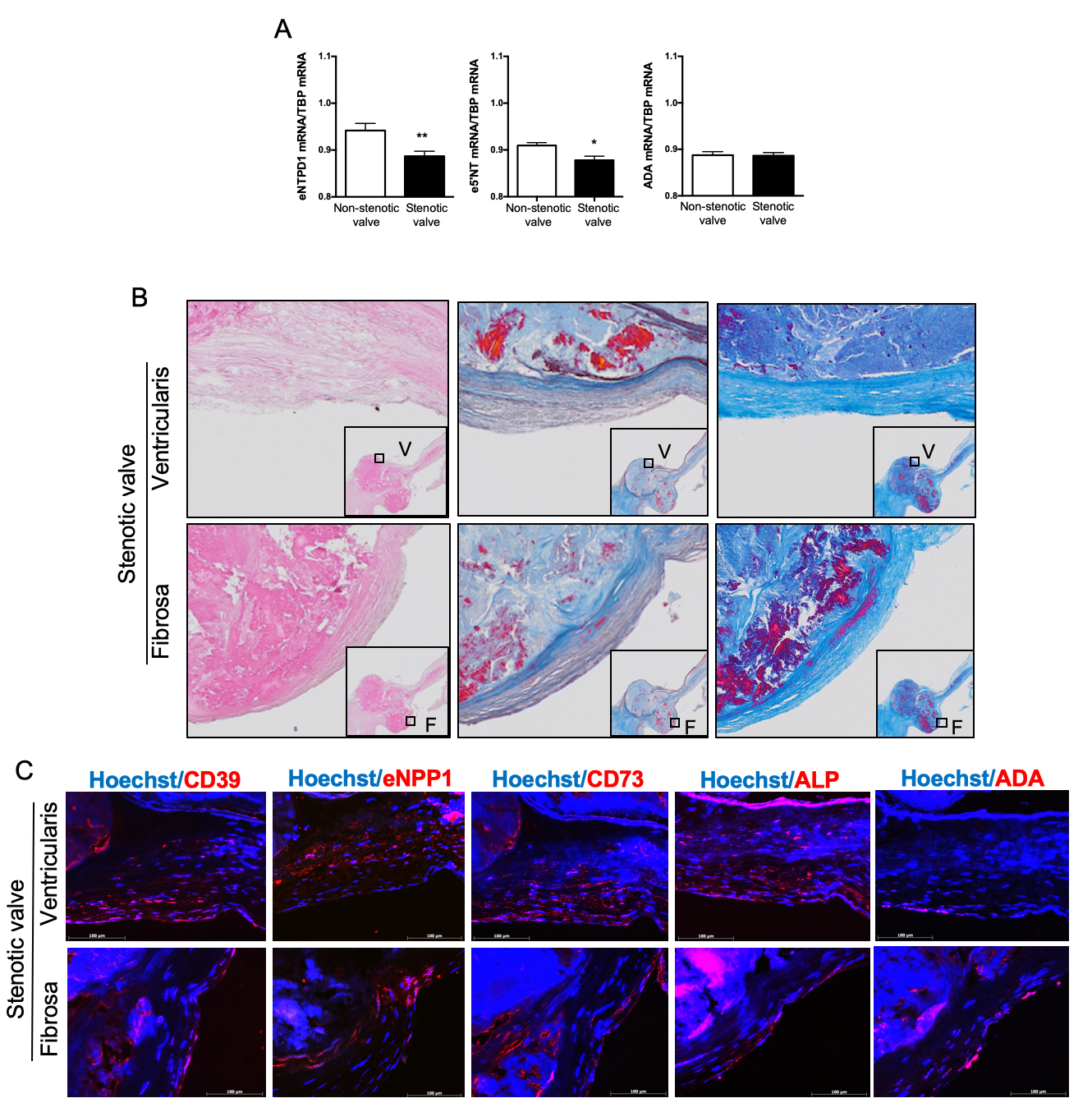


**Figure S3. The areas of calcification accumulates the signal for e5NT and ALP in immunofluorescence.** Relative mRNA expression for eNTPD1 (CD39), e5’NT (CD73) and ADA (adenosine deaminase) in human nonstenotic (n=6) and stenotic (n=9) aortic valves (**A)**. TBP mRNA were used for normalization. The average expression for each enzyme normalized per TBP mRNA was estimated from measurements for three leaflets independently, in the sites free of calcification. Results are shown as mean ± SD; **p*<0.01, ***p*<0.01 vs. non-stenotic valve by student *t*-test. Representative images of fibrosa and ventricularis of stenotic aortic valve (*n*=3) within regions of calcification **(B)** stained with Hematoxilin and Eosin (HE), Orcein Mertius Scarlet Blue (OMSB) and Masson’s Trichrome (TR). Scale bar = 100 μm. Representative images of matching sections stained by immunofluorescence (red signal) for CD39 (ecto-nucleoside triphosphate diphosphohydrolase 1), eNPP1 (ecto-nucleotide pyrophosphatase/ phosphodiesterase 1), CD73 (ecto5’-nucleotidase), ALP (alkaline phosphatase) and ADA (adenosine deaminase) **(C)**.

**
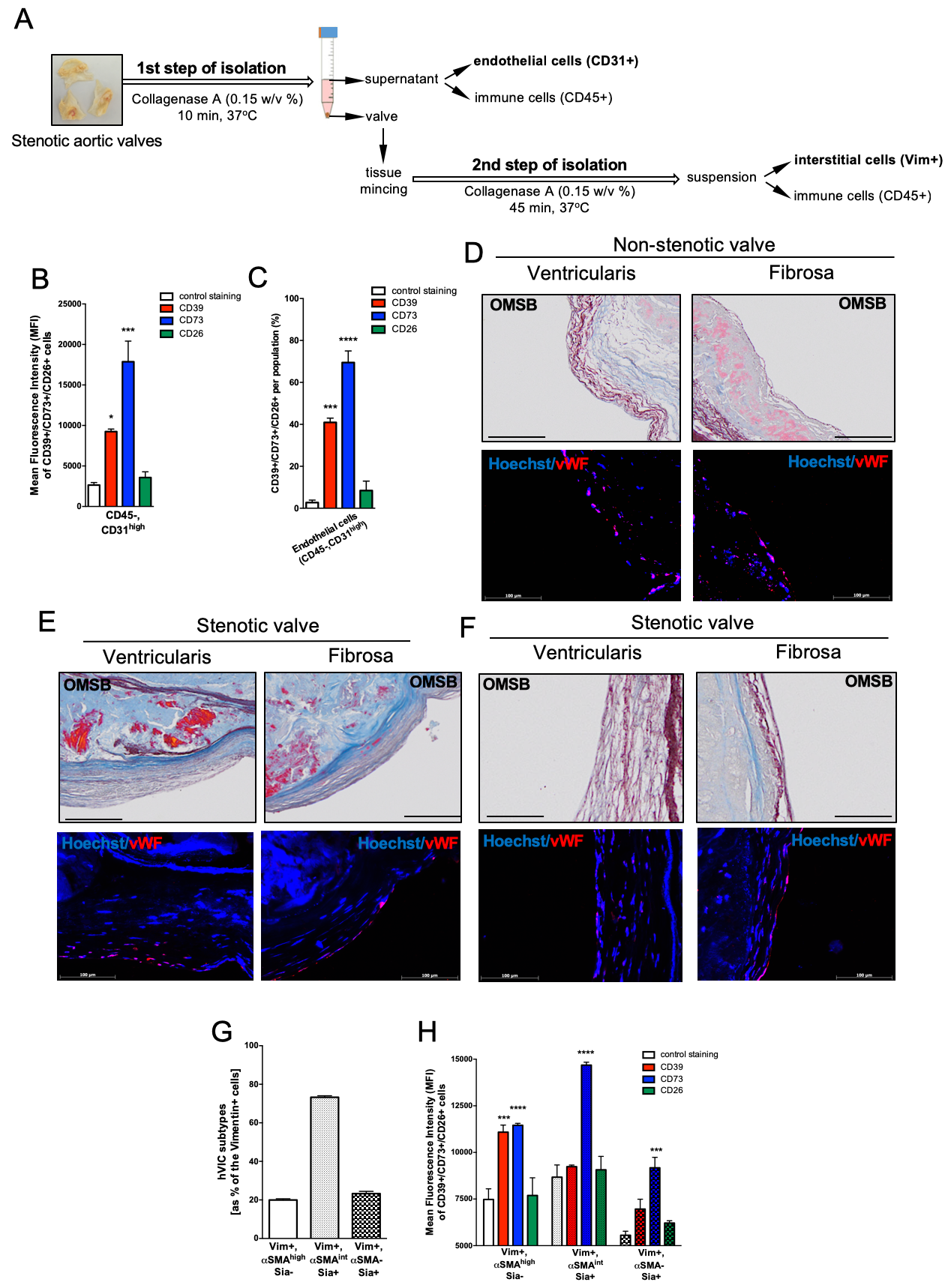
**

**Figure S4. Aortic valve endothelial and interstitial cells are the main source of nucleotide-degrading ecto-nucleotidases.** Simplified protocol stenotic aortic valve cell isolation **(A)**, including endothelial cells (1^st^ step of isolation, CD31^high^ positive) and interstitial cells (2^nd^ step of isolation; Vimentin positive, Vim+). Flow cytometry analysis **(B, C, G, H)**. Mean fluorescence intensity of cell-surface CD39, CD73 or CD26 (ADA-binding protein) for CD31^high^ positive endothelial cells **(B)**. CD39, CD73 or CD26 (ADA-binding protein) positive cells as a percentage (%) of total CD31^high^ positive endothelial cells isolated from stenotic aortic valve **(C)**. Results are shown as mean ± SEM; *n*=9 (independent isolations from 3 patients), **p*<0.05, ****p*<0.001, *****p*<0.0001 by one-way Anova followed by Holm-Sidak *post hoc* test. Representative images of fibrosa and ventricularis of non-stenotic **(D)** and stenotic **(E)** aortic valve (*n*=3) stained with Orcein Martius Scarlet Blue (OMSB) and representative images of matching sections stained by immunofluorescence (red signal) for vWF (von Willebrand factor). **(D)** Scale bar = 100 μm. Representative images of fibrosa and ventricularis of stenotic aortic valve (*n*=3) within regions of calcification **(E)** stained with OMSB and representative images of matching sections stained by immunofluorescence (red signal) for vWF (von Willebrand factor). Scale bar = 100 μm. Percentage of interstitial cells (Vim+) as myofibroblast like-interstitial cells (αSMA^high^/Sia-), myo-/osteoblast-like interstitial cells (αSMA^int^/Sia+) and osteoblast-like interstitial cells (αSMA-/Sia+) **(G)** and mean fluorescence intensity of cell-surface CD39, CD73 or CD26 (ADA-binding protein) for each type of interstitial cells **(H)**. Results are shown as mean ± SEM; *n*=9 (independent isolations from 3 patients), ****p*<0.001, *****p*<0.0001 by one-way Anova followed by Holm-Sidak *post hoc* test.


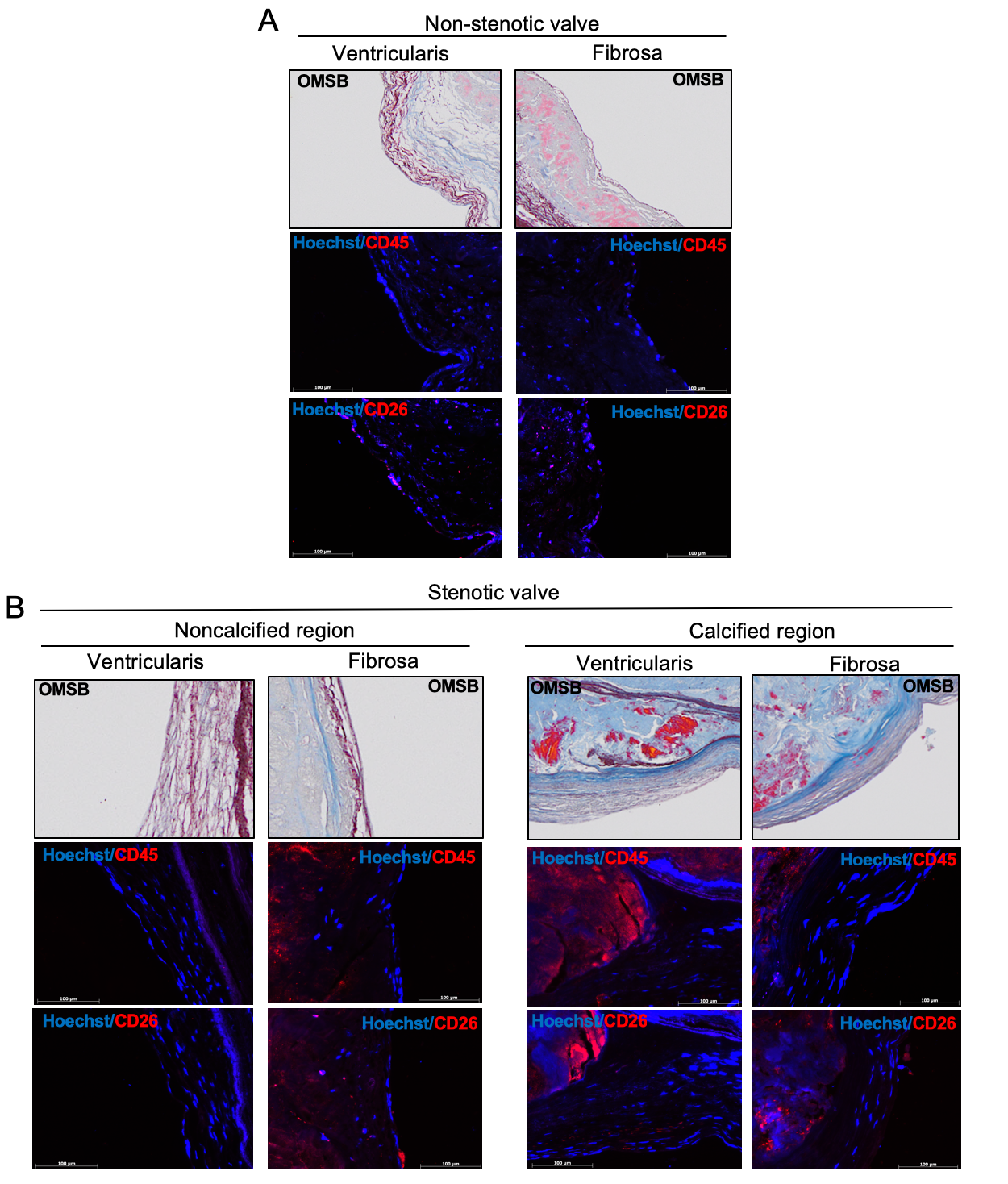


**Figure S5. Immune infiltrate localize in the fibrosa side of stenotic aortic valve and within the calcification regions.** Representative images of fibrosa and ventricularis of non-stenotic and stenotic aortic valve (*n*=3) stained with Orcein Mertius Scarlet Blue (OMSB) and representative images of matching sections stained by immunofluorescence (red signal) for CD45 and CD26 **(A)**. Representative images of fibrosa and ventricularis of stenotic aortic valve (*n*=3) in the regions free of calcification and within the calcification stained with OMSB and representative images of matching sections stained by immunofluorescence (red signal) for CD45 and CD26 **(B).** Scale bar = 100 μm.

**
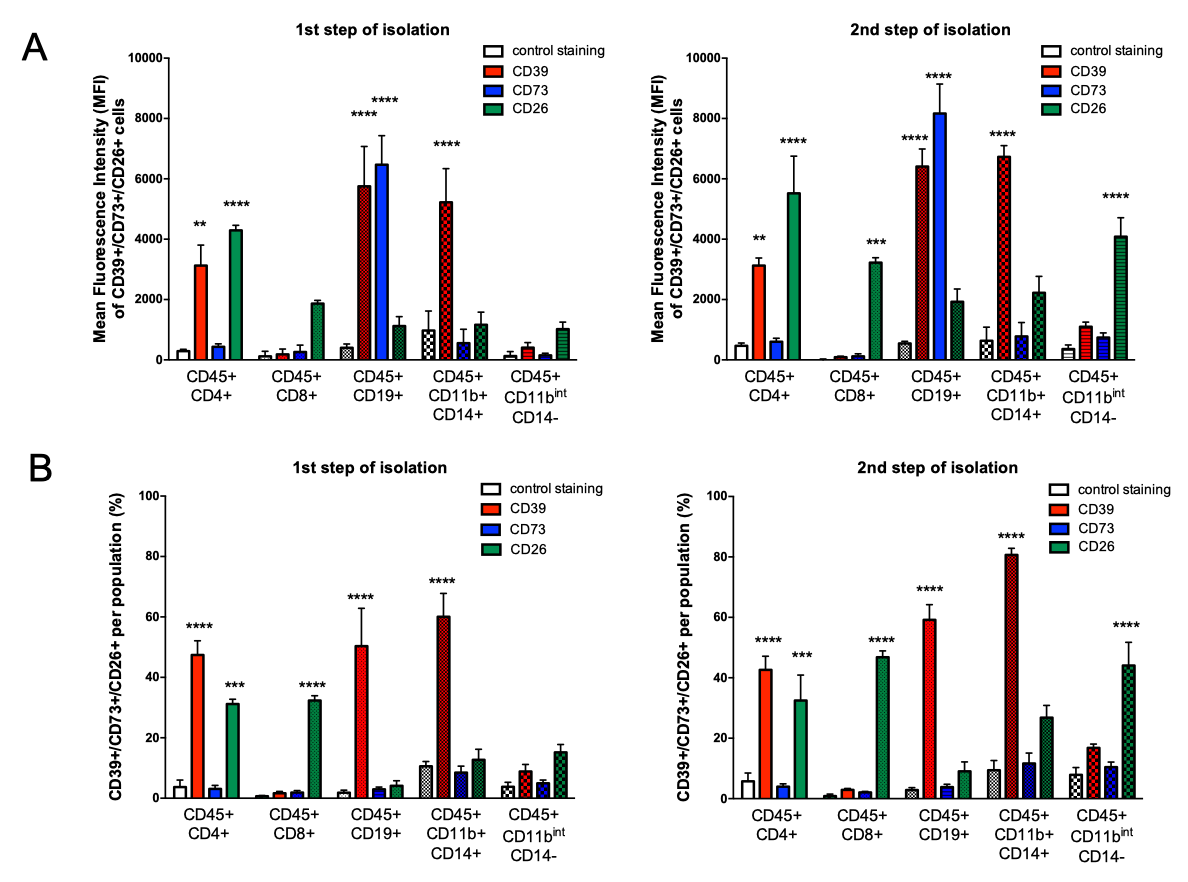
**

**Figure S6.** **Stenotic aortic valve immune infiltrate is a smaller source of nucleotide-degrading ecto-nucleotidases but a larger of adenosine deaminase.** Flow cytometry analysis. Mean fluorescence intensity of cell-surface CD39, CD73 or CD26 (ADA-binding protein) for each type of isolated immune cells (CD45+), including T helper cells (CD45+,CD4+), T cytotoxic cells (CD45+,CD8+), B cells (CD45+,CD19+), monocytes/macrophages (CD45+,CD11b+, CD14+) and granulocytes (CD45+,CD11b^int^, CD14-) **(A)**. CD39, CD73 or CD26 (ADA-binding protein) positive cells as a percentage (%) of each type of immune cells **(C)**. Results are shown as mean ± SEM; *n*=9 (independent isolations from 3 patients), ***p*<0.01, ****p*<0.001, *****p*<0.0001 vs. control staining **(B)** by Student *t*-test Holm-Sidak *post hoc* test.


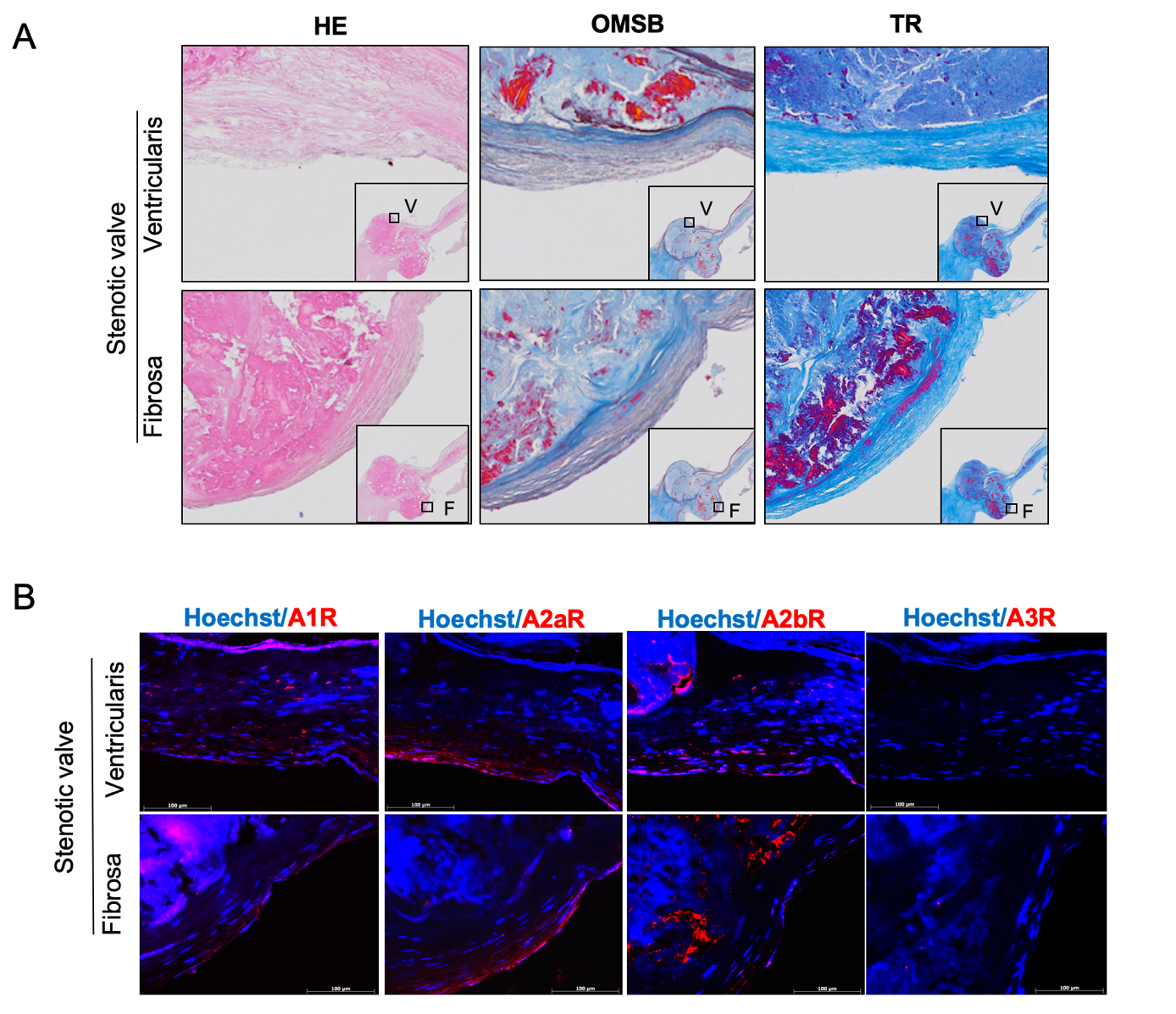


**Figure S7. Adenosine receptors are widely express in human non-stenotic and stenotic aortic valves.** Representative images of fibrosa and ventricularis of stenotic aortic valve (*n*=3) within regions of calcification stained with Hematoxilin and Eosin (HE), Orcein Mertius Scarlet Blue (OMSB) and Masson’s Trichrome (TR). Scale bar = 100 μm **(A)**. Representative images of matching sections stained by immunofluorescence (red signal) for four types of adenosine receptors **(B)**.
